# Tree rings provide a new class of phenotypes for genetic associations that foster insights into adaptation of conifers to climate change

**DOI:** 10.1111/nph.14968

**Published:** 2018-01-04

**Authors:** Johann M. Housset, Simon Nadeau, Nathalie Isabel, Claire Depardieu, Isabelle Duchesne, Patrick Lenz, Martin P. Girardin

**Affiliations:** ^1^ Natural Resources Canada Canadian Forest Service Laurentian Forestry Centre 1055 du P.E.P.S, PO Box 10380, Stn. Sainte‐Foy Québec QC G1V 4C7 Canada; ^2^ Natural Resources Canada Canadian Wood Fibre Centre 1055 du P.E.P.S, PO Box 10380, Stn. Sainte‐Foy Québec QC G1V 4C7 Canada; ^3^ Centre d’étude de la forêt Université du Québec à Montréal C.P. 8888, succ. Centre‐ville Montréal QC H3C 3P8 Canada; ^4^ Chaire de Recherche du Canada en Génomique Forestière Faculté de Foresterie de Géographie et de Géomatique Université Laval Québec QC G1V 0A6 Canada

**Keywords:** adaptive capacity, climate change, common garden, dendroecology, local adaptation, needleleaf, temperate forests, tree rings

## Abstract

- Local adaptation in tree species has been documented through a long history of common garden experiments where functional traits (height, bud phenology) are used as proxies for fitness. However, the ability to identify genes or genomic regions related to adaptation to climate requires the evaluation of traits that precisely reflect how and when climate exerts selective constraints.
- We combine dendroecology with association genetics to establish a link between genotypes, phenotypes and interannual climatic fluctuations. We illustrate this approach by examining individual tree responses embedded in the annual rings of 233 *Pinus strobus* trees growing in a common garden experiment representing 38 populations from the majority of its range.
- We found that interannual variability in growth was affected by low temperatures during spring and autumn, and by summer heat and drought. Among‐population variation in climatic sensitivity was significantly correlated with the mean annual temperature of the provenance, suggesting local adaptation. Genotype–phenotype associations using these new tree‐ring phenotypes validated nine candidate genes identified in a previous genetic–environment association study.
- Combining dendroecology with association genetics allowed us to assess tree vulnerability to past climate at fine temporal scales and provides avenues for future genomic studies on functional adaptation in forest trees.

## Abstract 2

See also the Commentary on this article by https://doi.org/10.1111/nph.15094.

## Introduction

In boreal and temperate ecosystems, a substantial increase in temperature and in the frequency of extreme events (e.g. severe droughts or heat waves) is projected for the upcoming century (IPCC, 2013). Poleward climatic niche shifts are expected (e.g. McKenney *et al*., 2014), therefore raising questions about the ability of trees to cope with those rapid changes (Price *et al*., 2013). Migration and long‐term genetic adaptation are expected to be too slow to keep pace with climatic niche shifts (Jump & Peñuelas, 2005; Aitken *et al*., 2008; Jump *et al*., 2009; Jezkova & Wiens, 2016). Nevertheless, populations may persist in their current location depending on species sensitivity and adaptive capacity to future environmental changes (Aubin *et al*., 2016). To better predict the fate of tree species, knowledge is needed about the climatic constraints affecting their growth/development and the key genetic and physiological mechanisms involved in the response to those constraints (Aubin *et al*., 2016; Urban *et al*., 2016). These knowledge gaps challenge the forecast of species’ responses to shifting climatic conditions and the implementation of forest management strategies such as assisted gene flow from pre‐adapted populations (Aitken & Whitlock, 2013). Without a genuine tree‐centered approach based on time‐series data to evaluate sensitivity to fluctuating environmental conditions, elucidating the genetic architecture of adaptation to climate throughout a tree's lifespan will remain difficult (Alberto *et al*., 2013).

Local adaptation in tree species has been documented through a long history of common garden experiments composed of georeferenced genetic material (provenances), where dendrometric traits such as diameter or height growth were used as proxies for fitness (Langlet, 1971; Alberto *et al*., 2013). The rapidity of climate change advocates the use of those existing common garden experiments (which have already been exposed to warming during the last few decades) to investigate responses to climate change (e.g. Rehfeldt *et al*., 1999; Sork *et al*., 2013). In combination with genomic approaches, common gardens represent a unique opportunity to decipher the genomic architecture of local adaptation, and to identify putative genes or genomic regions involved in tree species’ responses to climate (Lepais & Bacles, 2014; de Villemereuil *et al*., 2016).

Two genomic strategies are commonly used to search for genes involved in local adaptation to climate (Sork *et al*., 2013). The first is the genotype–environment association (GEA) method that looks for correlations between genetic markers and environmental variables from where populations/individuals originate (Joost *et al*., 2007; Coop *et al*., 2010). However, GEA does not provide information about the phenotypic traits controlled by the candidate genes. Another strategy is to test for associations between genotypes growing in a common environment and traits of interest (genotype–phenotype association; GPA). GPA has the advantage of establishing more direct links between genes and traits potentially under selection (e.g. Eckert *et al*., 2015). However, in tree species, the list of functional traits that closely reflect adaptation to climate is still rather limited and labor‐intensive to evaluate (for a review, see Aitken & Bemmels, 2016). In particular, assessment of dendrometric traits usually consists of punctual measurements (e.g. height or diameter at a certain age) that are probably the cumulative result of many different climatic events and constraints that occurred throughout the tree's lifespan. The use of innovative tools and methods to measure traits that reflect how and when climate exerts selective constraints (Rahaman *et al*., 2015; Urban *et al*., 2016) is thus needed to improve our understanding of species sensitivity and adaptive capacity to climate.

Through the investigation of interannual tree‐ring variations, dendroecology allows the quantification of climatic constraints that are exerted on trees. For decades, dendroecologists have developed methods to link climate with variations in wood anatomy characteristics (Fritts, 1976; Cook & Kairiūkštis, 1990; Schweingruber, 1996; Gessler *et al*., 2014; Housset *et al*., 2015; Girardin *et al*., 2016; Hartmann & Trumbore, 2016). Indeed, cambial activity is an integrative indicator that accounts for physiological mechanisms such as responses to drought stress, phenology of dormancy and resistance to freezing injuries. Those wood characteristics can be measured using nondestructive techniques to build a retrospective time‐series of growth‐related traits throughout a tree's lifespan. The influence of abiotic stress can be estimated by quantifying annual growth responses to punctual extreme climatic events (e.g. Montwé *et al*., 2016). Several recent reports have demonstrated the intraspecific genetic variation in growth response to punctual extreme climatic events (Montwé *et al*., 2016) or in cumulative growth responses to the average climate (over many years) in common garden experiments (McLane *et al*., 2011; Taeger *et al*., 2013; Leland *et al*., 2016). Most importantly, analysis of the relationships between growth time‐series and monthly climatic variables makes it possible to assess sensitivity to climatic constraints on a year‐to‐year basis. Therefore, it enables the identification of growth‐limiting factors and the time of the year (month or season) when trees are the most affected (Fritts, 1976; Girardin *et al*., 2012). Such tree‐ring traits reflecting sensitivity to climatic constraints have never been used as phenotypic traits in the context of quantitative genetics.

**Figure 1:**
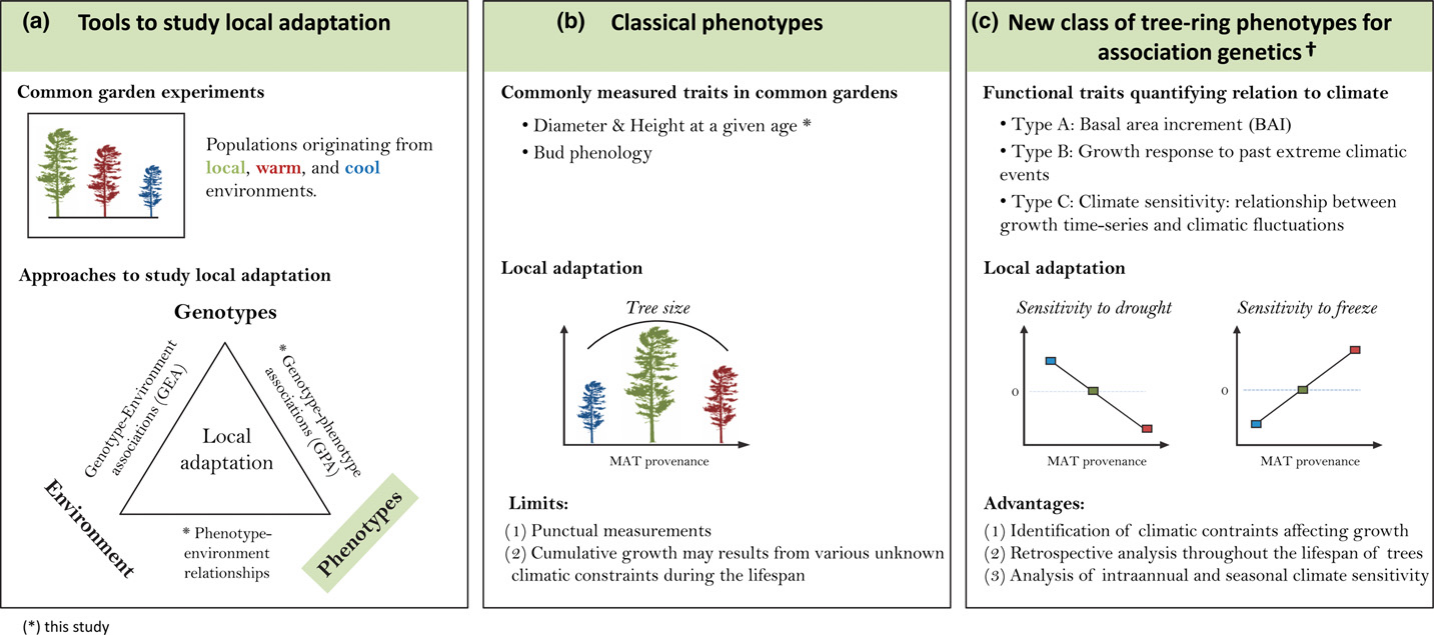
Tree‐ring trait assets in establishing the links between genotypes, phenotypes and environmental selection pressures acting on trees. (a) Tools to study local adaptation include common garden experiments and association genetics (triangle: adapted from Sork *et al*., 2013). (b) Commonly used phenotypes in dendroecology and forest genetics are based on dendrometric traits. (c) The use of tree‐ring phenotypes in association genetic studies allows fine temporal analysis of growth responses to past climatic events in addition to the identification of new candidate genes. †, Traits examined in the present study. Types A, B and C: please refer to the Materials and Methods section.

Here, we develop a new class of phenotypes that reflect responses to interannual climatic variability across trees’ lifespan. We then combine these new climate‐related phenotypes with association genetics to establish a link between the genotypes, phenotypes and climatic constraints affecting trees (Fig. 1). The present study demonstrates how tree‐ring traits derived from dendroecology can be used to identify and quantify climatic constraints influencing tree growth; study local adaptation in growth responses to such constraints; and identify and validate the genes putatively involved in such adaptation processes. We illustrate our approach using a *Pinus strobus* (eastern white pine, hereafter *P. strobus*) common garden experiment located in Quebec that includes provenances from a wide range of environments across the species’ distribution area in eastern North America. This species is representative of many temperate and boreal tree species for which the magnitude and speed of climate change are expected to induce widespread range shifts by 2060 (Joyce & Rehfeldt, 2013; Pedlar & McKenney, 2017). Local adaptation in *P. strobus* was made evident in provenance trial studies, which detected significant among‐population variation in classic quantitative traits (e.g. height, bud phenology: see Li *et al*., 1997; Joyce & Rehfeldt, 2013). More recently, GEA studies found several single nucleotide polymorphisms (SNPs) associated with climate (Nadeau *et al*., 2016; Rajora *et al*., 2016).

As a first step, a tree‐ring analysis was conducted to provide three different classes of phenotypic traits (Fig. 1). Traits included average annual ring width and density (hereafter type A traits), relative growth variation for a given year following an extreme climatic event (type B traits) and climatic sensitivity (CS) traits, that is, the strength of relationships between annual growth and climatic variation such as temperature and drought (type C traits). In a second step, those traits were correlated with mean annual temperature of the provenance (MAT_P_) to search for local adaptation in growth responses to climate (e.g. genetic–environment clines). Third, GPA analyses were performed on those tree‐ring traits to identify putative loci involved in growth responses to climatic constraints and to further cross‐validate candidate genes that had been identified in a previous GEA study (Nadeau *et al*., 2016).

## Materials and Methods

### Biological material and experimental design

**Figure 2:**
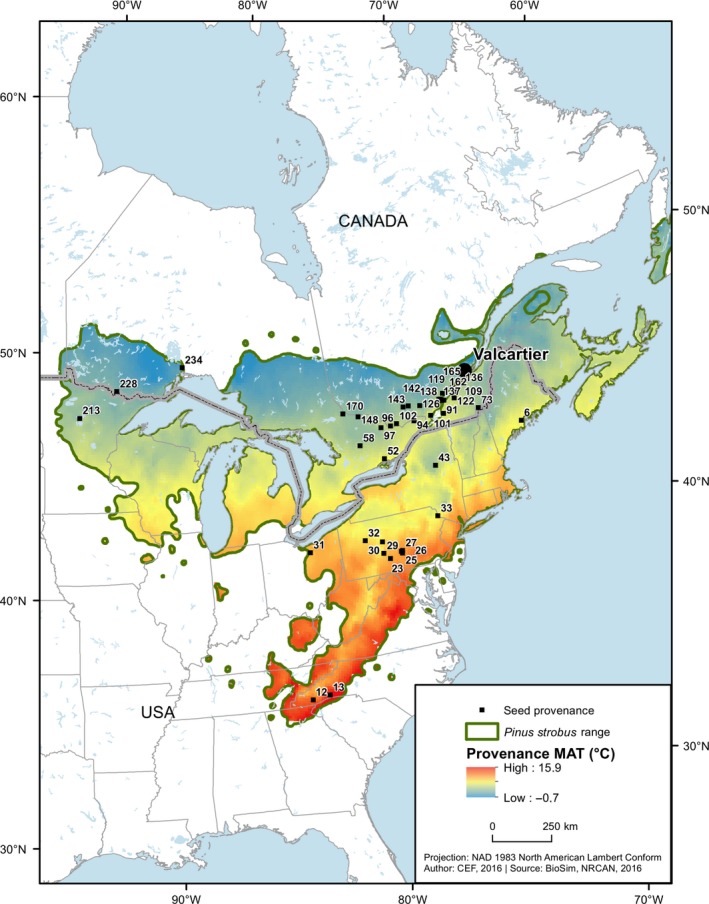
Location of the Valcartier common garden experiment and of provenances of sampled *Pinus strobus*. Mean annual temperature of each provenance (MAT_p_) averaged over 1993–2014 is superimposed on the range‐wide distribution of *P. strobus*.

Throughout this paper, we will use the term ‘provenance’ when referring to the geographic and climatic origin of a population, and the term ‘population’ when referring to the trees grown from seed sampled at the provenance (one population per provenance; McLane *et al*., 2011). A list of definitions for abbreviations used throughout this paper is given in Table 1. We studied *P. strobus* L. trees from 38 provenances planted in a common garden experiment. Provenances were selected to cover the majority of the species’ range from Canada and the US (Fig. 2; Supporting Information Table S1) to maximize the representativity of the intraspecific genetic variability. Nadeau *et al*. (2015) identified two main genetic groups (southern and northern) probably resulting from separate glacial refugia. Thirty‐six provenances were part of the northern genetic group and two provenances were from the southern group (provenances 12 and 13). Seeds were collected between 1976 and 1986 in natural stands, stored at −18°C, germinated in a glasshouse and cultivated for 3 yr in a tree nursery before their establishment in the common garden experiment at the Valcartier arboretum (Quebec, Canada; 46.94886°N, 71.4962°W) in 1989. The experiment was designed as two randomized complete blocks, with up to *n* = 16 trees per seedlot and blocks arranged in four‐tree row plots. Spacing between trees was 2.0 × 1.5 m (thinned to 2.0 × 3.0 m after the 2002 growing season) in block 1, and 2.0 × 2.0 m in block 2. On average, three trees (SD = 0.96) per population were sampled in each block. A total of 233 trees were sampled, representing two to ten trees per population (mean = 6.13, SD = 1.51), depending on the population survival rate (Table S1).

**Table 1:** Abbreviations used in this study

Abbreviation	Definition
BAI_*i*–*j*_	Basal area increment from year *i* to *j* (mm^2^ yr^−1^)
avDens_*i*–*j*_	Average density from year *i* to *j* (kg m^−^³)
D_21_	Diameter of 21‐yr‐old trees (m)
H_21_	Height of 21‐yr‐old trees (m)
GEA	Genotype–environment association
GPA	Genotype–phenotype association
DAPC	Discriminant analysis of principal components
CS_***X***_ **–** *Y*.*Z*_(*t*)_	Climatic sensitivity traits. Defined as the statistical associations between a growth metric ***X*** (residuals of BAI or avDens) and a climate variable *Y* (Temp for temperature; Prec for precipitation; Drought for drought code; Freeze for number of freezing days) in month *Z* (June of the year preceding growth (labelled *t*–1) to September of the year contemporaneous to growth (*t*))
MAP_p_	Mean annual precipitation of provenance (mm)
MAT_p_	Mean annual temperature of provenance (°C)
SNP	Single nucleotide polymorphism

### Meteorological data

Monthly means of daily temperature (*T*), mean monthly precipitation (*P*) and monthly means of daily drought code (DC) time‐series at the Valcartier experimental site were obtained for the 1993–2014 period using the BioSIM v.10 software (Régnière & Bolstad, 1994). Daily temperature and precipitation data were interpolated from the eight closest weather stations, adjusted for elevation and location differentials with regional gradients, and averaged using inverse distance weighting (1/*d* ^2^, where *d* is distance). Monthly values were computed from the average of the daily quantities. DC is an index for the amount of moisture stored in deep organic layers and soils and takes into account snow arrival in autumn and thawing in spring based on modeled snow accumulation (Terrier *et al*., 2013). The drought season is defined as the snow‐free period, which ranges from *c*. May to October. DC is unitless and ranges from 0 to *c*. 800, with low values indicating low drought risk and high values indicating high drought risk. Collinearity amongst these different monthly climate variables (temperature, precipitation and drought) is generally low according to pairwise Spearman rank correlation analysis (Table S2). Mean annual temperature and precipitation of each provenance (MAT_p_ and MAP_p_, respectively) were also computed and averaged over the 1961–1990 period (i.e. the period corresponding to seed collection) using BioSIM v.10.

### Tree‐ring data

At the end of October 2014, a 5‐mm‐diameter core was taken (from bark to pith) from each tree at 1.3 m above ground using a Pressler increment borer. Cores were kept frozen until being air‐dried, with no risk of developing mold. They were later sawn to a 1.7‐mm thickness longitudinally using a twin blade sleeve to obtain a smooth, thin surface. The same sample was used for dendrochronological and densitometric analyses. Annual rings of each core were visually cross‐dated with skeleton‐plots and pointer‐year identification under 4.5× magnification. Samples were then conditioned at a temperature of 20°C and relative humidity of 40% to maintain an equilibrium moisture content of 8%. For dendrochronological analyses, ring‐widths were measured from high‐resolution scanned images of the cores using the software Coo‐Recorder (Cybis, Saltsjöbaden, Sweden) at a precision of 10^−2^ mm. Cross‐dating was validated using the software CDendro (Cybis) and cofecha (Holmes, 1983). The ring‐width measurement series were converted into basal area increment (BAI) chronologies using the R library ‘dplR’ 1.6.3 (Bunn, 2008). Average annual BAI for the 1993–2014 period was calculated for each population from the raw data to obtain absolute levels of growth.

Two BAI metrics were used in downstream analyses: the raw – absolute BAI and standardized residual BAI scores. Eliminating the intrinsic age‐ and size‐related growth trends in BAI makes it possible to address annual growth variability independently of biological age or tree size. Here, raw BAI chronologies, which were not normally distributed, were transformed using a logarithmic scaling and detrended using modified generalized exponential functions (‘Hugershoff’ type; Warren, 1980) with the software arstan v.41d XP (Cook & Holmes, 1986). Additionally, an autoregressive model was applied to remove temporal autocorrelation in the time‐series, and residuals were extracted. Annual averages of these standardized residual BAI scores were computed for each population using a robust bi‐weight mean, which removes outliers attributable to disturbance or endogenous site factors (R library ‘dplR’ 1.6.3; Bunn, 2008). The mean interseries correlation (rbar) computed from all of the standardized residual tree‐level BAI time‐series (Wigley *et al*., 1984) was rbar = 0.80 (SD ± 0.17). The expressed population signal obtained for the residual tree‐level series was 0.99, which was higher than the commonly used threshold value (0.85) for growth–climate correlation analysis (Wigley *et al*., 1984).

The density of each thin core slice was measured using a Quintek X‐ray measuring system at Université Laval (Quebec, Canada). Cores were scanned in 0.04‐mm steps, producing high‐resolution density profiles. Average annual density was determined for each tree ring and the correct identification of annual ring borders was verified using the previously cross‐dated ring‐width measurements. Mean ring density was calculated for each population by averaging the annual ring density of all trees in a population for the 1993–2014 period. Then, for each core, average ring‐density time‐series were detrended using modified generalized exponential functions and temporal autocorrelation was removed as described above. Finally, annual averages of these standardized residual density scores were computed for each population as described above.

### Three classes of tree‐ring traits derived using a dendroecological approach

**Figure 3:**
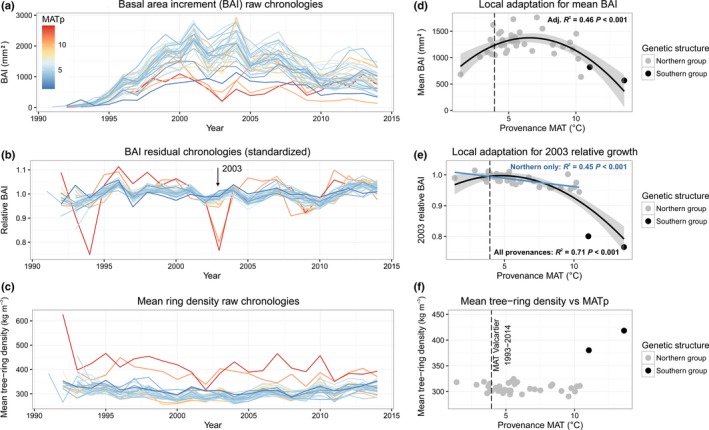
Tree‐ring phenotypes. (a) Annual raw–absolute basal area increment (BAI), (b) annual standardized residual BAI scores and (c) annual raw‐absolute tree‐ring density averaged by populations. The color gradient represents the mean annual temperature of the provenance (MAT_p_, °C) (red, warmer; blue, colder). The following relationships between MAT_p_ and tree‐ring phenotypes of the populations are illustrated: (d) BAI averaged over 1993–2014, (e) relative growth variation in 2003 (BAI_2003_) and (f) tree‐ring density averaged over 1993–2014. The genetic structure groups described by Nadeau *et al*. (2015) are represented by gray (northern group) and black (southern group) points. Significant relationships among all provenances suggesting local adaptation are represented by black lines. Significant relationships for the subset of provenances from the northern genetic structure group are represented by a blue line. The relationships were chosen according to a model selection procedure (Akaike information criterion, see the Materials and Methods section) between a linear model and a second‐order polynomial regression model. Only significant models are shown.

Traits were classified according to the following nomenclature (A, B and C; see description in Fig. 1c): (A) mean xylem BAI (meanBAI_1993–2014_) and average density (avDens_1993–2014_) over the 1993–2014 period, corresponding to classic measures used in forestry; (B) growth variation for a specific year following an extreme climatic event; and (C) climatic sensitivity (CS) traits. Type A traits are long‐term averages that probably result from many climatic events or constraints on growth throughout a tree's lifespan, whereas type B and type C traits reflect growth responses to climate on a year‐to‐year basis. To illustrate type B traits, we analyzed the standardized residual BAI scores for the year 2003 (BAI_2003_) because we observed large among‐population variation during that year (Fig. 3), which could be related to lower temperatures observed in autumn 2002 and spring 2003 (Figs S1, S2). The standardized residual BAI_2003_ scores indicate the relative growth variation of a population in comparison to its average growth level. Therein, a value of BAI_2003_ < 1 indicates a lower growth in 2003 as compared with the population's average growth level for the 1993–2014 period or, conversely, a BAI_2003_ > 1 indicates a higher growth than average.

CS (type C) traits were determined as follows. For each population, correlation analyses were computed between the annual standardized residual BAI scores, averaged at the population level, and the matrix of monthly climatic data (from 1993 to 2014) interpolated at the location of the common garden (Biondi & Waikul, 2004). Long‐term trends in climatic series were linearly detrended to obtain unbiased data of interannual climatic variations. Correlation analyses were performed for the period spanning from June of the year preceding ring formation to September of the year concurrent with ring formation to account for lagged effects from year to year (e.g. previous autumn freezing injury). The coefficients of correlation between growth metrics and climatic variables hereafter constitute the CS traits. Significance in correlation analyses was assessed using bootstrapped randomization (*n *=* *1000 simulations): when the 95% confidence interval included zero, the null hypothesis of ‘no relationship’ could not be rejected. CS traits were also calculated at the individual‐tree level to be used as phenotypes in GPAs (see below). As is routinely done in dendroecology, a response function analysis was also used as a complement to quantify the influence of climate on tree growth (Cook & Kairiūkštis, 1990). This analysis is a multiple regression technique that uses the principal components of monthly climatic data to estimate growth–climate associations. This complementary analysis, made in support of the correlation analysis, controls for collinearity of monthly climate predictors; results are presented in the Supporting Information section and are not discussed herein (Figs S3, S4). All analyses were conducted using the R library ‘treeclim’ 1.0.11 (Zang & Biondi, 2015). CS traits of the average density metric were also determined by repeating all of the above analyses on the annual standardized residual density scores, averaged at the population level.

### Local adaptation in growth responses to climate

We screened tree‐ring traits (type A, B and C) exhibiting among‐population variation that could be correlated with the climate of the provenance, an indication of local adaptation in growth responses to climate (Sork *et al*., 2013). We used MAT_p_ as an indicator because it was highly correlated with the number of growing degree‐days above 5°C (*r *=* *0.97), which was found to be the best predictor of genetic variation in growth potential among *P. strobus* populations (Joyce & Rehfeldt, 2013). MAT_p_ is correlated with MAP_p_ in our study system (Spearman *r *=* *0.42, with *P *=* *0.030 and df = 24, taking into account spatial autocorrelation; Dutilleul, 1993), and hence it also partially reflects a moisture availability gradient. First, Mantel tests were conducted to determine if similarities between population‐averaged time‐series of standardized residual BAI scores, as measured using pairwise correlations between time‐series, and geophysical characteristics of the provenances, were related (‘mantel.rtest’ function in the ade4 R package; Dray & Dufour, 2007). Next, for type A and B traits, the relationship with provenance MAT_p_ was tested using both linear and second‐degree polynomial regressions. We tested for the effect of block sample size by including a covariate in these models, which was defined as the sample size ratio of block 1 vs block 2; the effect of the covariate was not significant. Model selection was based on the Akaike information criterion (Burnham & Anderson, 2010). The null hypothesis (*H*_0_) was ‘no relationship with MAT_p_’. Regarding the effect of MAT_p_ on CS traits (type C), a linear regression model provided the best fit. To reduce the risk of generating spurious results, we only tested the CS traits for which growth–climate correlations were significant in at least five populations. This threshold was arbitrarily set to allow sufficient interpopulation variance in the CS traits for the detection of genetic associations.

### Genetic data acquisition

For genotyping, DNA from each of the 233 sampled trees was extracted from foliage using the protocol described by Nadeau *et al*. (2015). Leaf sampling was conducted independently from the sampling of Nadeau *et al*. (2015, 2016): only 21 out of 133 populations sampled in Nadeau *et al*. (2015, 2016) are common to both datasets (16%), and samples within populations are largely different (*c*. 4% in common). The 233 trees were genotyped on the same set of 153 SNP markers as in Nadeau *et al*. (2015) previously developed in a Sequenom iPlex Gold MassARRAY (Agena Bioscience, San Diego, CA, USA). This array included SNPs from 52 candidate genes for growth and phenology previously identified in *Picea glauca* and 68 noncandidate genes randomly distributed across the genome (for more details see Nadeau *et al*., 2015, 2016). Using this array and an independent set of populations distributed across the range of *P. strobus*, Nadeau *et al*. (2016) identified 26 candidate SNPs for local adaptation using GEAs and *F*_ST_ outlier tests. In this study, eight trees and six SNPs failed during the genotyping step and were discarded from subsequent analyses. SNPs with a minor allele frequency < 0.03 were also discarded. This resulted in 225 trees and 128 SNPs successfully genotyped (SNP genotyping call rate: min. = 90.7%, mean = 98.9%), including 21 of the candidate SNPs identified by Nadeau *et al*. (2016). All of the 21 candidate SNPs were detected by at least one GEA method, and two SNPs were *F*_ST_ outliers (Nadeau *et al*., 2016; Table S3). No trees were discarded based on the genotyping call rate (min. = 78.9%; mean = 98.9%). GenBank accession numbers of the genes containing the 128 SNPs are given in Table S3.

### Genotype–phenotype associations

We searched for genes involved in growth‐sensitivity to climate by performing GPA analyses on tree‐ring traits. GPAs were tested at the individual‐tree level for the 128 SNPs using tassel v.5.0 software (Yu *et al*., 2006; Zhang *et al*., 2010). We only tested the tree‐ring traits that showed a highly significant correlation with MAT_p_ at the population level (*P *<* *0.01, see Results). tassel tests for associations between genotypes and phenotypes for each SNP–trait combination. The full linear mixed model used can be expressed as follows:

y=Xβ+Sα+Qv+Zμ+ϵ,

where y is the vector of observations for a tree‐ring trait (i.e. the phenotypes), β is the vector of fixed block effects, α is the vector of fixed SNP (genotypes) effects, v is the vector of fixed population structure effects (described below), μ is a vector of additive or ‘background’ polygenic effects, which takes into account relatedness among individuals, and ϵ is the vector of random residuals. *X*,* S*,* Q* and *Z* are incidence matrices relating y to β, α, v and μ, respectively. Random additive effects and errors are assumed to be normally distributed with μ ~ *N*(0, 2*K* $\sigma_{a}^{2}$) and ϵ ~ *N*(0,*I* $\sigma_{\epsilon }^{2}$), where $\sigma_{a}^{2}$ is the additive genetic variance, $\sigma_{\epsilon }^{2}$ is the residual variance, *K* is the kinship matrix and *I* is the identity matrix. The kinship matrix *K* was computed from the 128 SNPs using the method of Ritland (1996) implemented in the program SPAGeDi v1.5a (Hardy & Vekemans, 2002).

Population structure can create spurious associations between genotypes and phenotypes (false positives). Nevertheless, when population structure covaries with adaptive genetic variation as in *P. strobus* (Nadeau *et al*., 2016), over‐adjustment for population structure may decrease power and result in false negatives (Sork *et al*., 2013). Therefore, three models were tested with tassel: no correction (i.e. omitting the population structure and kinship effects); correction for population structure (***Q*** model, i.e. omitting the kinship effects); and correction for population structure and for kinship (***Q*** + ***K*** model, i.e. full model). To obtain the population membership matrix (***Q***), we performed a discriminant analysis of principal components (DAPC; Jombart *et al*., 2010) using all 225 genotyped trees from this study and 821 additional trees (133 provenances) sampled across the entire range and genotyped on the same set of 128 SNPs taken from Nadeau *et al*. (2015). DAPC maximizes among‐population genetic variation while minimizing within‐group genetic variation. Therefore, this method is well suited for GPA analyses because population structure is removed by including the group memberships identified by DAPC as covariates in the tassel model (Jombart *et al*., 2010). A complete description of the DAPC method can be found in Fig. S5. We used the membership probabilities for *k* = 3 clusters (*k *− 1 = 2 covariates) as the ***Q*** matrix to correct for population structure in the tassel analyses (Fig. S5b). The three clusters detected were similar to those in Nadeau *et al*. (2015): a southern and a northern group with weak west to east differentiation (Fig. S5c). To summarize results from the three models (no correction, ***Q*** and ***Q*** + ***K***), each SNP–phenotype association was classified as follows: ‘very likely’ when significant (*P *<* *0.05) for all three models; ‘likely’ when significant for two out of the three models; ‘uncertain’ when significant for only one model; and ‘no evidence’ when not significant in the three models (Girardin *et al*., 2016). While summarizing results across models may help controlling for errors, the likelihood of false discoveries is significantly raised under multiple testing. Because we tested a small number of SNPs (128), we preferred to reduce the risk of false negatives (i.e. not detecting true associations) by using *P*‐values that were not corrected for multiple testing. Instead, the cross‐validation of the SNPs detected by tassel with the 21 candidate SNPs found in Nadeau *et al*. (2016), which used independent samples and different methods, should reduce false positives and make a stronger case for those candidate genes. GPAs excluding the two southern group populations (*n *=* *221) gave similar results (Table S4).

To look for functional categories, genes containing SNPs involved in significant GPAs were annotated using blastx against the RefSeq database (https://www.ncbi.nlm.nih.gov/refseq) using a threshold *E*‐value of 1 e^−10^. Given that two *P. strobus* sequences had no significant hit on the RefSeq database, blast analyses were performed against the *P. glauca* gene catalog (tblastx,* E*‐value of 10^−10^; Gcat 3.3; Rigault *et al*., 2011) and the *P. strobus* transcriptome (blastn,* E*‐value of 10^−20^; Ingo Ensminger (University of Toronto), personal communication). Complementary information regarding their putative function was then deduced from *P. glauca* and *P. strobus* best‐ortholog TAIR annotations (TAIR, *Arabidopsis thaliana* database, https://www.arabidopsis.org/index.jsp). SNP annotations (synonymous, nonsynonymous) were deduced as previously described by Nadeau *et al*. (2016).

## Results

High variability was observed for annual averages of raw‐absolute BAI among *P. strobus* populations (Fig. 3a). Globally, time‐series of population‐averaged standardized residual BAI scores were well intercorrelated; the mean of the pairwise correlations was 0.66. Yet, the values of these pairwise correlations tended to be higher when computed between provenances that had smaller geographic distances (Mantel test observed correlation *r *= −0.30, *P *=* *0.019) and smaller MAT_p_ differences (*r *= −0.39, *P *=* *0.003).

### CS traits

**Figure 4:**
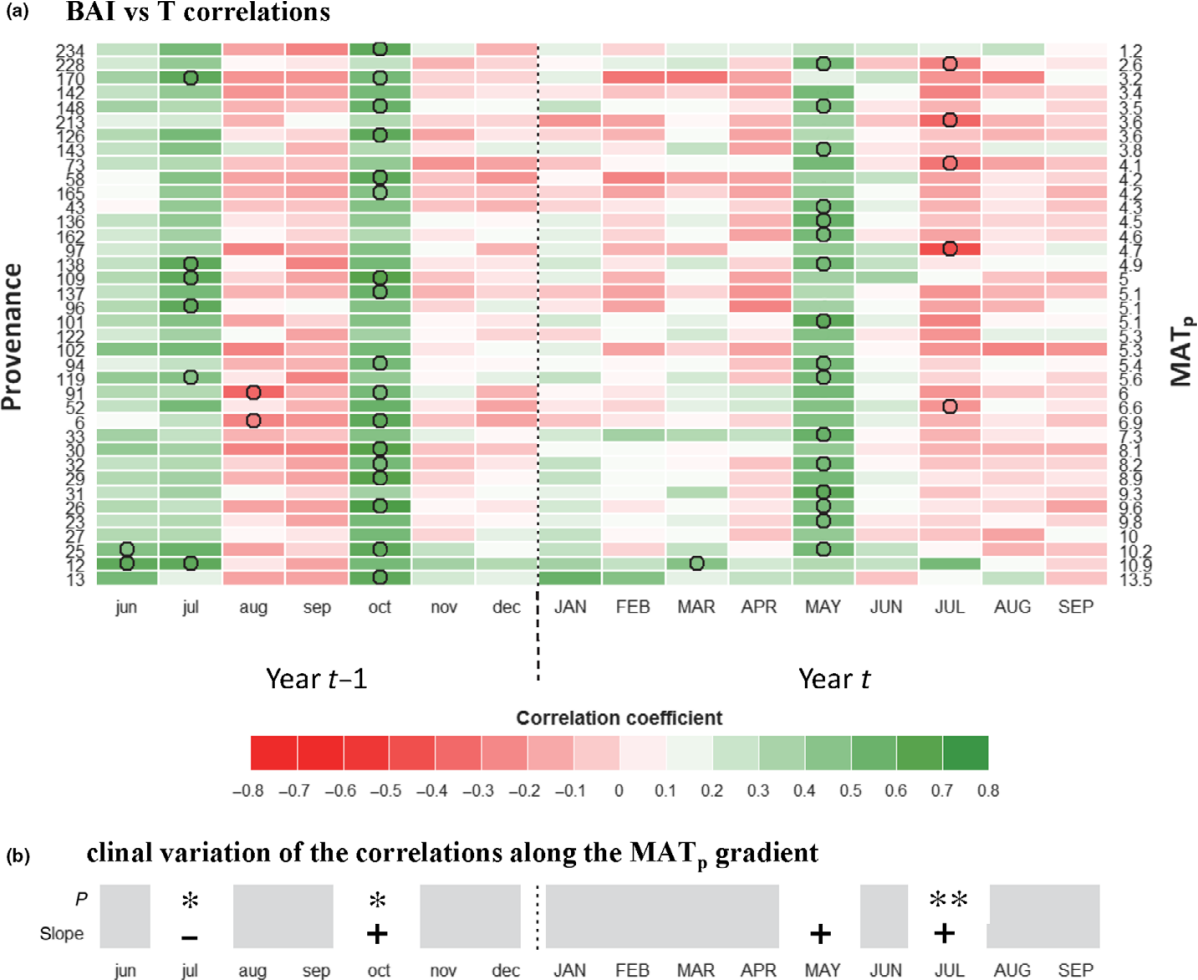
Climatic sensitivity (CS) traits: basal area increment (BAI) and monthly temperature (T). (a) Bootstrapped correlation coefficients were computed between *Pinus strobus* standardized residuals of BAI scores (from Fig. 3b) and monthly mean temperature at the Valcartier common garden experiment (Quebec, Canada) over the 1993–2014 period. Populations (rows) are ordered by increasing mean annual temperature of the provenance (MAT_p_; right axis labels). Months in capital letters represent the current year of ring formation; months in lower case represent climate variables during the year preceding ring formation. Significant coefficients are represented by circles. (b) Test for clinal variations in correlation coefficients along the MAT_p_ gradient: *P*‐value and slope sign of a regression against MAT_p_. Significance: *, *P *<* *0.05; **, *P *<* *0.01.

**Figure 5:**
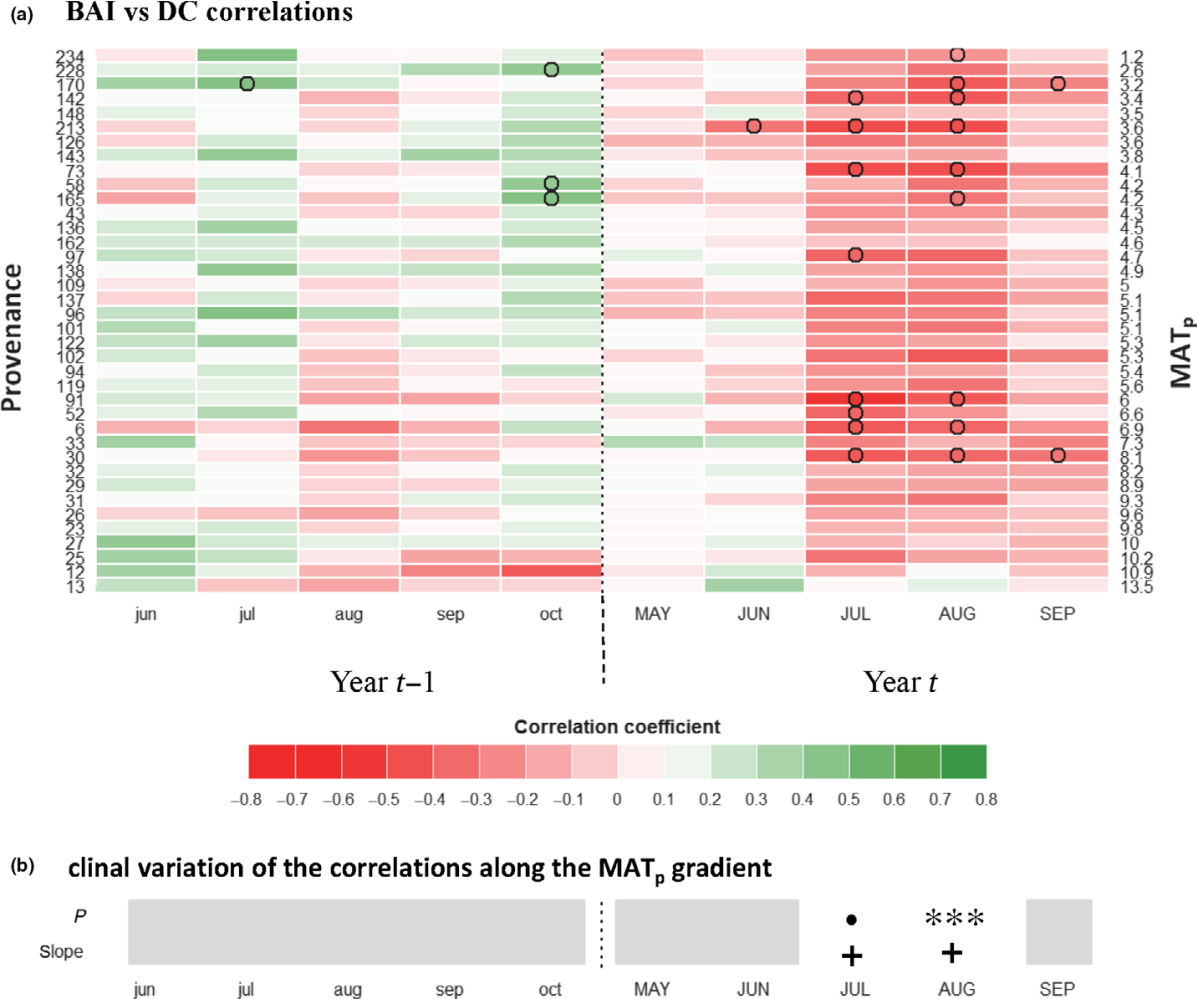
Climatic sensitivity (CS) traits: basal area increment (BAI) and monthly drought code (DC). (a) Bootstrapped correlation coefficients were computed between *Pinus strobus* BAI standardized residuals of BAI scores and the monthly DC at the Valcartier common garden experiment (Quebec, Canada) over the 1993–2014 period. (b) Test for clinal variations in correlation coefficients along the MAT_p_ gradient: *P*‐value and slope sign of a regression against MAT_p_. Significance: •, *P *<* *0.1; ***, *P *<* *0.001.

Analysis of interannual variability of population‐averaged standardized residual BAI and density scores in relation to climatic fluctuations enables the identification of climatic constraints acting upon ring formation. The coefficients of correlation between growth metrics and the climatic variables hereafter constitute the CS traits (type C; Figs 4, 5; see Figs S3, S4 for response function analyses). The positive influence of a lengthening of the growing season on BAI was made evident by the positive correlations between population averages of standardized residual BAI scores (1993–2010) and October temperatures during the autumn of the year preceding ring formation (CS_BAI_–Temp.Oct_(*t−*1)_), and with May temperature of the year concurrent with ring formation (CS_BAI_–Temp.May_(*t*)_) (Fig. 4a). Furthermore, standardized residual BAI scores were negatively correlated with the numbers of freezing days occurring in October of the year preceding growth (CS_BAI_–Freeze.Oct_(*t*−1)_) and in June of the year of ring formation (CS_BAI_–Freeze.Jun_(*t*)_) (Fig. S6a). In addition, the effect of heat stress on BAI was revealed by the negative correlations between standardized residual BAI scores and July temperature of the current growth year (CS_BAI_–Temp.Jul_(*t*)_; Fig. 4a). Negative correlations with July and August DCs (CS_BAI_–Drought.Jul_(*t*)_ and CS_BAI_‐Drought.Aug_(*t*)_) were also observed, highlighting the influence of summer drought stress on BAI (Fig. 5a). In parallel, standardized residual BAI scores were positively correlated with current‐year July precipitation (CS_BAI_–Prec.Jul_(*t*)_; Fig. S7a). Individual‐tree level CS traits were consistent with the CS traits computed at the population level (visual inspection, Fig. S8). As for ring density, a negative effect of summer drought during the year preceding growth was detected (Fig. S9). Tree‐ring density was positively influenced by high temperatures during the growing season (May, June and July; Fig. S10).

### Local adaptation in tree‐ring traits

The raw BAI averaged by population over the entire period (absolute meanBAI_1993–2014_, type A trait), expressed as a function of MAT_p_, exhibited a second‐order curve (Fig. 3d; adj *R* ^2^ = 0.46, *P *<* *0.001). This revealed a high degree of variation among populations and suggested local adaptation to the mean annual temperature of the provenance. According to that model, the best‐performing population was nonlocal. The MAT_p_ of the population that had the highest growth performance was 6.6°C, which is higher than the MAT in the Valcartier common garden experiment (3.95°C). Populations from both warmer and colder provenances exhibited lower growth. No significant relationship was observed between absolute avDens_1993–2014_ and MAT_p_ (Fig. 3c). However, note that the two populations from the southern group have a much higher tree‐ring density (Fig. 3f).

Among‐population variation was also observed for climate‐related tree‐ring traits (types B and C). The reduction in growth in 2003 (BAI_2003_, type B trait) was stronger for the populations from warmer provenances and absent in some cold‐climate provenances (Fig. 3e). Growth in 2003 was particularly reduced for the two southernmost provenances, corresponding to the southern genetic group. Among‐population genetic variation was found for CS traits using growth–climate correlations (type C traits). Among traits related to heat and drought stresses, CS_BAI_–Temp.Jul_(*t*)_ (Fig. 4) and CS_BAI_–Drought.Aug_(*t*)_ (Fig. 5) were positively correlated with MAT_p_, and CS_BAI_–Prec.Jul_(*t*)_ (Fig. S7) was negatively correlated with MAT_p_. Among traits reflecting cold tolerance, CS_BAI_–Freeze.Oct_(*t*−1)_ and CS_BAI_–Freeze.Jun_(*t*)_ (Fig. S6) were negatively correlated with MAT_p_, whereas CS_BAI_–Temp.Oct_(*t*−1)_ (Fig. 4) was positively correlated with MAT_p_.

### CS and genotype–phenotype associations

We searched for genes related to local adaptation in growth responses to climate by testing those tree‐ring traits that showed a significant (*P *<* *0.01) correlation with MAT_p_: meanBAI_1993–2014_ (type A), BAI_2003_ (type B) and five CS traits (type C): CS_BAI_–Drought.Aug_(*t*)_, CS_BAI_–Prec.Jul_(*t*)_, CS_BAI_–Temp.Jul_(*t*)_, CS_BAI_–Freeze.Oct_(*t*−1)_ and CS_BAI_–Freeze.Jun_(*t*)_. Diameter at breast height (DBH_21_) and height measured at age 21 (H_21_), referred to as dendrometric phenotypic traits in the present study, were also tested. GPA analysis revealed significant associations (*P *<* *0.05) with dendrometric traits, as well as with the three types of tree‐ring traits for both candidate and noncandidate SNPs (Table S3). For dendrometric traits (DBH_21_ and H_21_) and type A tree‐ring traits (meanBAI_1993–2014_), the proportion of significant SNPs was similar between candidate and noncandidate SNPs (Table S5). For all the climate‐related type B (BAI_2003_) and type C traits (CS traits), we found a greater proportion of significant candidate SNPs as compared to noncandidate SNPs, although the proportion of SNPs classified as very likely was greater for noncandidate SNPs for CS_BAI_–Prec.Jul_(*t*)_ and CS_BAI_‐FreezeOct_(*t*−1)_.

**Figure 6:**
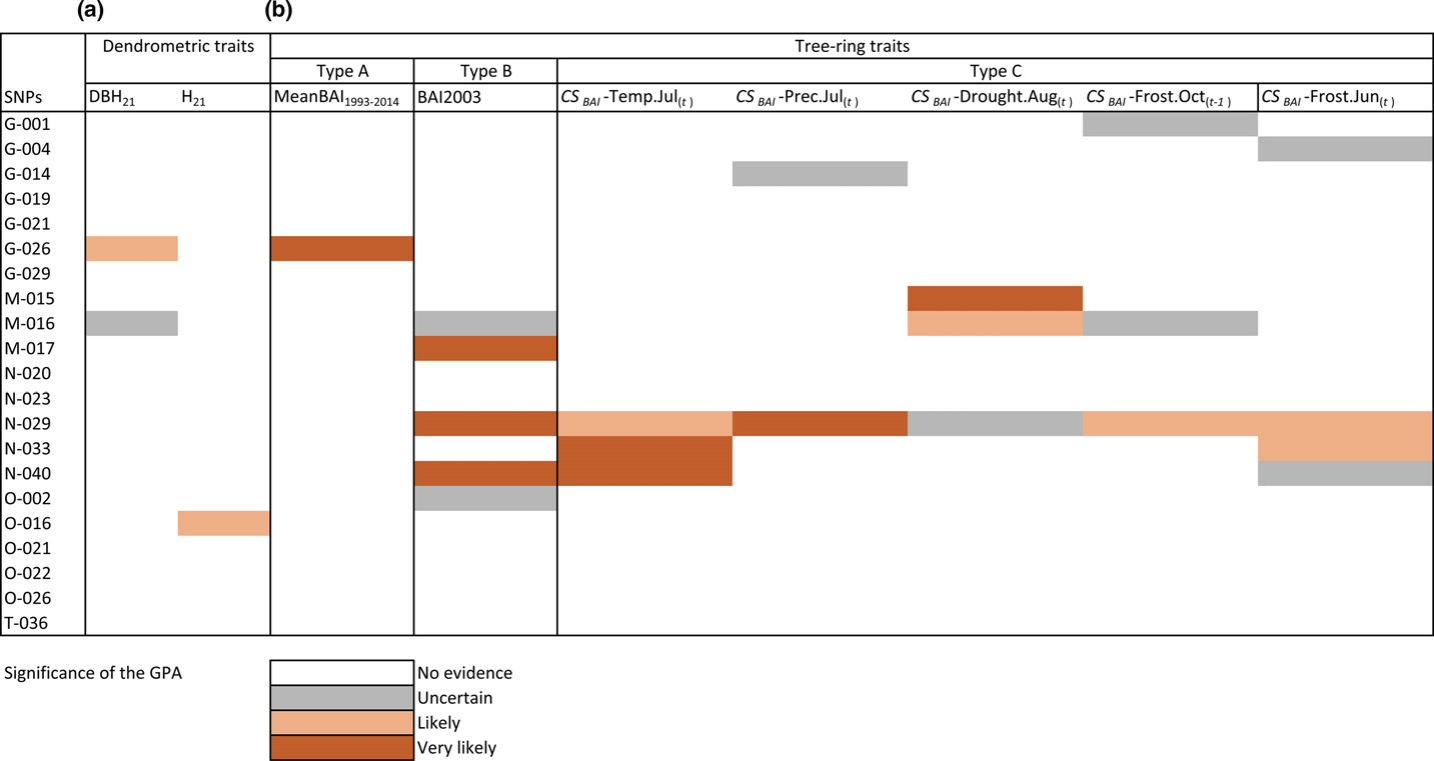
Tests of genotype–phenotype associations (GPAs) between traits and candidate loci from a previous genotype–environment association (GEA) analysis (Nadeau *et al*., 2016): (a) dendrometric traits (DBH_21_ and H_21_ = diameter at breast height and height at 21 yr), and (b) tree‐ring traits of types A, B and C. For each SNP–trait pair, the association was tested with different levels of corrections: without correction, with correction for the genetic structure (***Q***), and with correction for both genetic structure and kinship (***Q*** + ***K***). The significance of the associations was classified as very likely (when significant for three levels of correction), likely (when significant for two levels) or uncertain (when significant for only one level of correction). BAI, basal area increment; CS, climate sensitivity trait; Temp, temperature; Prec, precipitation; Drought, drought code; Freeze, number of freezing days during the month.

Among the 21 candidate SNPs tested (Nadeau *et al*., 2016), 12 were confirmed in this study (10 genes; Fig. 6; Table 2): three SNPs (three genes) were significantly associated with dendrometric traits DBH_21_ and H_21_, while 11 SNPs (nine genes) were associated with tree‐ring traits (types A, B and C). As many as 10 SNPs (eight genes) were associated with the climate‐related type B and type C traits (seven SNP–trait associations classified as very likely, five likely and eight uncertain). Traits reflecting responses to specific freezing events (BAI_2003_, CS_BAI_–Freeze.Oct_(*t*−1)_, and CS_BAI_–Freeze.Jun_(*t*)_) were associated with SNPs G‐001, G‐004, M‐016, M‐017, N‐029, N‐033, N‐040 and O‐002. CS traits reflecting heat and drought tolerance (CS_BAI_–Temp.Jul_(*t*)_, CS_BAI_–Prec.Jul_(*t*)_, CS_BAI_–Drought.Aug_(*t*)_) were associated with SNPs G‐014, M‐015, M‐016, N‐029, N‐033 and N‐040.

**Table 2:** Annotation of the candidate single nucleotide polymorphisms (SNPs) associated with dendrometric and tree‐ring traits

Trait type	SNP	Gene	SNP annotation^a^	Annotation^b^	Traits pointed out by GPA	Known function	Known response to abiotic stresses
Dendrometric	O‐016	2_6731_01	NS	SLY1 gene, F‐box family protein	H_21_	Positive regulator of GA signaling in *Arabidopsis thaliana* (Ariizumi *et al*., 2011)	GA_3_ in *A. thaliana* (Ariizumi *et al*., 2011)
Dendrometric, A	G‐026	GQ0206.B3_C13	NS	Hypothetical protein ACMD2_05917 (*Ananas comosus*)	DBH_21_, meanBAI_1993–2014_	–	–
B	M‐017	0_8844_01	Intron	Galacturonosyltransferase (GAUT) 14	Tolerance to cold: BAI_2003_	Carbohydrate metabolism and cell wall pectin biosynthetic processes in *A. thaliana* (Sterling *et al*., 2006; Wang *et al*., 2011)	Cold in conifers (Bannister & Neuner, 2001)
B	O‐002	0_8844_01	S	Galacturonosyltransferase (GAUT) 14	Tolerance to cold: BAI_2003_	Carbohydrate metabolism and cell wall pectin biosynthetic processes in *A. thaliana* (Sterling *et al*., 2006; Wang *et al*., 2011)	Cold in conifers (Bannister & Neuner, 2001)
C	G‐004	GQ0033.TB_H23	Intron	Endoglucanase 25‐like	Tolerance to cold: CS_BAI_–Frost.Jun_(*t*)_	Carbohydrate metabolism and cellulose synthesis (*A. thaliana*,* Picea glauca*; Maloney, 2010)	Diverse abiotic stresses in herbaceous plants (Wang *et al*., 2016)
C	G‐001	GQ0015.B3.r_B10	Intron	Vacuolar‐processing enzyme beta‐isozyme 1‐like	Tolerance to cold: CS_BAI_–Frost.Oct_(*t*−1)_	Storage protein accumulation (Gruis *et al*., 2004)	Stress‐induced programmed cell death process (Hatsugai *et al*., 2015)
C	G‐014	GQ0081.BR.1_D09	NS	Plastid movement impaired 1‐related 1 (PMIR1)	Tolerance to drought: CS_BAI_–Prec.Jul_(*t*)_	Chloroplast photorelocation movement (Suetsugu *et al*., 2015)	Drought in *A. thaliana* (Rojas‐Pierce *et al*., 2014)
C	N‐033	0_7001_01	NS	NADPH‐dependent diflavin oxidoreductase 1‐like isoform X3	Tolerance to cold: CS_BAI_–Frost.Jun_(*t*)_ Tolerance to heat: CS_BAI_–Temp.Jul_(*t*)_	Modulation of plant growth and development in higher plants (Lodeyro *et al*., 2012; Karlusich *et al*., 2014)	Diverse abiotic stresses using transgenic plants (Lodeyro *et al*., 2012)
C	M‐015	0_8683_01	S	Serine/threonine‐protein kinase At1g18390	Tolerance to drought: CS_BAI_–Drought.Aug_(*t*)_	Signaling and plant defense (Afzal *et al*., 2008)	Diverse abiotic stresses in herbaceous plants (Afzal *et al*., 2008)
Dendrometric, B, C	M‐016	0_8683_01	NS	Serine/threonine‐protein kinase At1g18390	DBH_21_ Tolerance to cold: BAI_2003_, CS_BAI_–Frost.Oct_(*t*−1)_ Tolerance to drought: CS_BAI_–Drought.Aug_(*t*)_	Signaling and plant defense (Afzal *et al*., 2008)	Diverse abiotic stresses in herbaceous plants (Afzal *et al*., 2008)
B, C	N‐029	0_6047_02	Not annotated	Basic helix–loop–helix (bHLH) DNA‐binding superfamily protein	Tolerance to cold: BAI_2003_, CS_BAI_–Frost.Oct_(*t*−1)_, CS_BAI_–Frost.Jun_(*t*)_ Tolerance to heat: CS_BAI_–Temp.Jul_(*t*)_ Tolerance to drought: CS_BAI_–Prec.Jul_(*t*)_, CS_BAI_–Drought.Aug_(*t*)_	Anthocyanin biosynthesis, cell proliferation and differentiation (Yamada *et al*., 2011; Xiang *et al*., 2015)	Diverse abiotic stresses (Ji *et al*., 2016)
B, C	N‐040	2_4107_01	S	Thylakoid lumenal 19 kDa protein, chloroplastic	Tolerance to cold: BAI_2003_, CS_BAI_–Frost.Jun_(*t*)_ Tolerance to heat: CS_BAI_–Temp.Jul_(*t*)_	Photosynthetic electron transfer (Ishihara *et al*., 2007)	Diverse abiotic stresses in herbaceous plants (Gururani *et al*., 2015)

Known functions and responses to abiotic stresses of homologous genes in other plant species are reported in the two right‐hand columns of the table.

–, No information reported.

a S, synonymous SNP; NS, nonsynonymous SNP.

b The most informative annotation has been selected among the 10 top hits for the GQ0081.BR.1_D09 gene.

The annotation of the 12 candidate SNPs confirmed in this study revealed a good match with the known functions of homologous genes in other plant models (Table 2). Eight SNPs were located in coding regions and three were located in a noncoding region (intron). Among those located in coding regions, three SNPs were identified as synonymous and five were identified as nonsynonymous. All the putative gene products had functions ranging from transcription factor activity to regulation of signaling molecules.

## Discussion

This study illustrates how the combined analysis of tree‐ring traits derived from dendroecology and genotypes from a common garden experiment can contribute to understanding the complex relationship between genotypes, phenotypes (growth responses embedded in the annual rings) and interannual climatic fluctuations. Indeed, the new class of phenotypes obtained provided useful information to identify climatic constraints for a given tree species. We illustrated this approach by an examination of the CS traits shaping the growth of an important conifer in eastern North America's temperate forests. We quantified the sensitivity of populations to climatic‐related constraints across their lifespan and identified the critical times of the year (or previous year) when those constraints affected growth. We then highlighted the clinal variation along MAT_p_ for those traits. This approach made it possible to identify candidate genes for local adaptation in growth responses to climatic variability, and to cross‐validate candidate loci obtained from an independent GEA study.

### Tree sensitivity to climate

The dendroclimatic analysis pointed to traits reflecting CS for each population (type C traits). At our study site, growth was mainly limited by very low/freezing temperature during the dehardening phase in spring and cold acclimation phase in autumn, when trees are probably the most vulnerable (probably causing freeze injuries; Howe *et al*., 2003); and by summer heat and drought stress.

The positive influence of a longer growing season on growth is shown by the positive effect of previous October and current May temperatures (Fig. 4), as well as the negative effect of previous October and current June number of days below 0°C (Fig. S6). Warm spring conditions result in an earlier cessation of dormancy and earlier cambium reactivation, which leads to a longer growth period (Begum *et al*., 2013). Similarly, a warm autumn during the year before ring formation translates into more time available for accumulation of nonstructural carbohydrate reserves (Hartmann & Trumbore, 2016) and for the division of leaf primordia meristematic cells in buds (Fritts, 1976; Macey & Arnott, 1986). This influence of temperature in combination with photoperiod on the growth of the tree populations under study could be confirmed by studying bud and cambium phenology (e.g. Perrin *et al*., 2017).

Growth was limited by summer heat or drought, with a negative correlation with July temperature (Fig. 4) and July and August drought severity (Fig. 5), and a positive correlation with July precipitation (Fig. S7). Warm summers can have a direct negative effect on growth by increasing the ratio of carbohydrates allocated to maintenance respiration, while hydric stress reduces both water intake and photosynthesis through stomata closure (Hartmann & Trumbore, 2016). The correlations with climate that we found for this common garden were consistent with those reported for natural stands in other regions (e.g. Marchand & Filion, 2011; Chhin *et al*., 2013; Girardin *et al*., 2016).

The study of tree rings makes it possible to precisely determine the influence of yearly climatic anomalies on growth during a tree's life cycle; this information cannot be obtained from conventional approaches used in tree genetics, such as the study of tree height or diameter at a given age. It also has the advantage of accounting for time‐lagged effects of climate, which is particularly relevant for conifer species showing a determined growth pattern. For instance, autumn temperatures may influence the timing of budburst of the subsequent year, which in turn can impact tree fitness (Heide, 2003).

### Tree adaptation potential to climate change

Comparing the populations’ responses to climate (type B and type C traits) in the common garden revealed existing genetic variation for tolerance to freeze and drought. The positive influence of a warm autumn during the year preceding ring formation (CS_BAI_–Temp.Oct_(*t*−1)_) was stronger for populations from southernmost provenances and significantly increased with MAT_p_ (Fig. 4a,b). Furthermore, the negative effect of early autumn freeze (CS_BAI_–Freeze.Oct_(*t*−1)_) and late spring freeze (CS_BAI_–Freeze.Jun_(*t*)_) was greater for warm provenances (Fig. S6). The timing of bud set and bud flush are two important traits involved in the adaptation to local climate that are known to be under strong genetic control in forest trees (e.g. Billington & Pelham, 1991; Savolainen *et al*., 2004; Bennie *et al*., 2010; McKown *et al*., 2014). Populations from colder provenances have probably evolved to set buds and enter dormancy earlier, thereby minimizing the risk of early autumn freeze injuries. Clinal variations in cold hardiness were also reported for *P. strobus* (Lu *et al*., 2003), thus supporting our findings that warmer provenances were more likely to be impacted by freeze injuries and to exhibit reduced growth. Genetic variation related to cold hardiness was further demonstrated by among‐provenance variations in the standardized residual growth of 2003 (BAI_2003_) following lower than average temperatures (e.g. −2°C) during autumn 2002 and spring 2003 (type B traits). The relative growth reduction following this event was significantly more pronounced for populations from warmer provenances (Fig. 3e), with the two southern group provenances exhibiting a very severe growth reduction.

Local adaptation in response to drought was suggested by the correlation between MAT_p_ and among‐population variation of CS_BAI_–Temp.Jul_(*t*)_ (Fig. 4b), CS_BAI_–Drought.Aug_(*t*)_ (Fig. 5b) and CS_BAI_–Prec.Jul_(*t*)_ (Fig. S7). Provenances from warmer locations were less sensitive to summer drought in the common garden, suggesting that those provenances were more adapted to droughts (in agreement with Savva *et al*., 2002; McLane *et al*., 2011; Taeger *et al*., 2013; Montwé *et al*., 2016). Our study demonstrates the great potential of using CS derived from tree rings sampled in common gardens to investigate trees’ adaptive capacity to climate change, especially when testing provenances for suitability in future warming climates.

### Genetic basis of climate sensitivity

GPA analysis conducted on a combination of dendrometric and tree‐ring traits allowed the detection of significant associations for both candidate and noncandidate SNPs from Nadeau *et al*. (2016). While the noncandidate SNPs may also contain relevant genes for responses to climate, our main goal was to see whether we could confirm those candidate SNPs that had previously been detected in genetic–environment associations (Nadeau *et al*., 2016) and gain further insights about their putative roles in adaptation to climate. For climate‐related traits (types B and C), we found a greater proportion of significant SNPs in candidate than in noncandidate SNPs. In addition, the independent validation of candidate SNPs from a previous study using different samples and different methods should reduce false positives and so we further discuss the candidate genes here. GPA analyses confirmed 12 of the 21 candidate SNPs (six very likely, two likely, four uncertain), and 10 of them (eight genes) were associated with climate‐related traits (types B and C). The five strongest candidate SNPs in Nadeau *et al*. (2016; namely N‐029, G‐014, M‐015, M‐016 and M‐017), which had previously been detected by at least two GEA or *F*_ST_ outlier methods, were also significantly associated with climate‐related tree‐ring traits in this study. Although false positives are possible, many loci were associated with several traits and with different levels of correction for population structure and kinship, thus increasing our confidence in the results.

The associations between candidate SNPs and type B and C tree‐ring traits provided information about the climatic constraints affecting *P. strobus* growth, and thus helped bridge the gap between the genotypes, the phenotypes and the environmental selection pressures. Taken together, our results suggest that the putative functions obtained for the genes detected in GPA associations were plausible, because information found in the literature supports the established associations between the candidate SNPs and the measured traits reflecting either drought tolerance, freeze tolerance or both. Several SNPs were associated with both freeze and drought tolerance traits (three very likely and one likely; Fig. 6). Among those, SNP N‐029 was located within a bHlH (basic helix–loop–helix) transcription factor, which is known to confer tolerance to multiple abiotic stresses in higher plants (Table 2). Similarly, the gene containing SNP N‐040 codes for a thylakoid luminal protein involved in photosystem II, one of the most susceptible components of the photosynthetic machinery that bears the brunt of abiotic stresses. Other SNPs were specifically associated with either freeze or drought tolerance traits. Interestingly, the GAUT (SNPs M‐017, very likely; O‐002, uncertain) and the endoglucanase genes (SNP G‐004, uncertain), which are involved in carbohydrate metabolism, were specifically associated with growth responses to freezing events (Table 2). By preserving and stabilizing cell structures, carbohydrates associated with the cell wall can enhance freezing tolerance by controlling water movement and ice formation under cold conditions (Tenhaken, 2015). In addition, one SNP (G‐014; uncertain), exclusively associated with drought tolerance traits (Table 2), encoded a PLASTID MOVEMENT IMPAIRED1 (PMI1), a protein that mediates the abscisic acid (ABA)‐dependent pathway under drought conditions in *Arabidopsis thaliana* (Rojas‐Pierce *et al*., 2014). Contrary to cold‐responsive genes, many water‐stress‐inducible genes are upregulated by ABA under drought conditions (Yamaguchi‐Shinozaki & Shinozaki, 2006). Further gene expression and functional studies aimed at identifying coexpressed gene networks involved in specific biological processes, such as freeze tolerance, would be needed to determine the exact role of those genes in *P. strobus* and in other conifers (for a review, see Serin *et al*., 2016). Multi‐SNP GPA models (e.g. Segura *et al*., 2012) should also be tested given that highly metric traits such as growth are probably controlled by a large number of interacting genes, which was beyond the scope of this study given the small number of SNPs tested.

### Conclusion

Accurately determining the periods of the year when trees are most vulnerable to abiotic and biotic factors is crucial to understanding the genetic architecture of local adaption. In this paper, we showed that tree‐ring traits measured in common garden experiments offer great potential to unravel local adaptation in forest trees through a fine‐scale, retrospective and dynamic view of tree responses to past environmental variations. For the first time, we proposed using a metric (i.e. climate sensitivity traits; CS) that incorporates both the phenotypic trait under study (i.e. growth or wood density) and the response to climate in association genetic studies. We identified important climatic constraints and the critical times of the year when those constraints affect growth, information that cannot be derived from classic measurements such as height and DBH. We believe that this novel approach will provide a valuable contribution to the study of differences in climate sensitivity between provenances in common garden experiments, and more generally to examine forest adaptation to environmental changes.

## Author contributions

J.M.H., S.N., C.D., I.D., P.L., N.I. and M.P.G. planned and designed the research; J.M.H., S.N., M.P.G. and C.D. performed the experiments and analyzed the data; J.M.H., S.N., C.D., I.D., P.L., N.I. and M.P.G. wrote the manuscript. J.M.H. and S.M. contributed equally to this work.

## Supporting information

Please note: Wiley Blackwell are not responsible for the content or functionality of any Supporting Information supplied by the authors. Any queries (other than missing material) should be directed to the *New Phytologist* Central Office.

**Fig. S1** Monthly mean temperature, drought code, minimum temperature and precipitation in 2002 and 2003 at Valcartier, compared to interannual variability during the 1993–2014 period.
**Fig. S2** Spring mean temperature and August drought code in 2003, and number of days with minimum temperature below zero in October 2002 at Valcartier, compared to interannual variability in standardized residual BAI scores.
**Fig. S3** Correlation analysis for climatic sensitivity traits basal area increment and monthly temperature.
**Fig. S4** Correlation analysis for climatic sensitivity traits basal area increment and monthly drought code.
**Fig. S5** Genetic structure assessed through a discriminant analysis of principal components (DAPC).
**Fig. S6** Correlation analysis for climatic sensitivity traits basal area increment and monthly number of days below 0°C.
**Fig. S7** Correlation analysis for climatic sensitivity traits basal area increment and monthly precipitation.
**Fig. S8** Comparison of growth–climate relationships at the provenance and at the individual levels.
**Fig. S9** Correlation analysis for climatic sensitivity traits ring average density and monthly drought code.
**Fig. S10** Correlation analysis for climatic sensitivity traits ring density and monthly mean temperature.
**Table S1** Summary of provenance location, mean annual temperature of provenance, mean annual precipitation and number of sampled trees per provenance
**Table S2** Spearman rank correlations between monthly climate variables
**Table S5** Comparisons between the number of SNPs detected in GPAs with tassel between candidate and noncandidate SNPs

**Table S3** Detailed results of the genotype–phenotype association (GPA) analysis with tassel

**Table S4** Detailed results of the genotype–phenotype association (GPA) analysis with tassel when excluding the two populations from the southern group
