# Supplementary material for: Tree rings provide a new class of phenotypes for genetic associations that foster insights into adaptation of conifers to climate change
Source: New Phytol. 2018 Jan 4;218(2):630–45. doi: 10.1111/nph.14968 (PMC6079641; doi:10.1111/nph.14968)
Supplement: Supplementary file 1 — Fig. S1 Monthly mean temperature, drought code, minimum temperature and precipitation in 2002 and 2003 at Valcartier, compared to interannual variability during the 1993–2014 period. Fig. S2 Spring mean temperature and August drought code in 2003, and number of days with minimum temperature below zero in October 2002 at Valcartier, compared to interannual variability in standardized residual BAI scores. Fig. S3 Correlation analysis for climatic sensitivity traits basal area increment and monthly temperature. Fig. S4 Correlation analysis for climatic sensitivity traits basal area increment and monthly drought code. Fig. S5 Genetic structure assessed through a discriminant analysis of principal components (DAPC). Fig. S6 Correlation analysis for climatic sensitivity traits basal area increment and monthly number of days below 0°C. Fig. S7 Correlation analysis for climatic sensitivity traits basal area increment and monthly precipitation. Fig. S8 Comparison of growth–climate relationships at the provenance and at the individual levels. Fig. S9 Correlation analysis for climatic sensitivity traits ring average density and monthly drought code. Fig. S10 Correlation analysis for climatic sensitivity traits ring density and monthly mean temperature. Table S1 Summary of provenance location, mean annual temperature of provenance, mean annual precipitation and number of sampled trees per provenance Table S2 Spearman rank correlations between monthly climate variables Table S5 Comparisons between the number of SNPs detected in GPAs with tassel between candidate and noncandidate SNPs [file NPH-218-630-s001.pdf]

New Phytologist

***New Phytologist* Supporting Information**

Article title: Tree rings provide a new class of phenotypes for genetic associations that foster insights into adaptation of conifers to climate change

Authors: Johann M. Housset, Simon Nadeau, Nathalie Isabel, Claire Depardieu, Isabelle Duchesne, Patrick Lenz, Martin P. Girardin
Article acceptance date: 21 November 2017

The following Supporting Information is available for this article:

Fig. S1 Monthly mean temperature, drought code, minimum temperature and precipitation in 2002 and 2003 at Valcartier, compared to inter-annual variability during the 1993-2014 period.

Fig. S2 Spring mean temperature and August drought code in year 2003, and number of days with minimum temperature below zero in October 2002 at Valcartier, compared to inter-annual variability in standardized residual BAI scores.

Fig. S3 Correlation analysis for climatic sensitivity traits basal area increment and monthly temperature.

Fig. S4 Correlation analysis for climatic sensitivity traits basal area increment and monthly drought code.

Fig. S5 Genetic structure assessed through a discriminant analysis of principal components (DAPC).

Fig. S6 Correlation analysis for climatic sensitivity traits basal area increment and monthly number of days below 0 ºC.

Fig. S7 Correlation analysis for climatic sensitivity traits basal area increment and monthly precipitation.

Fig. S8 Comparison of growth-climate relationships at the provenance and at the individual levels.

Fig. S9 Correlation analysis for climatic sensitivity traits ring average density and monthly drought code.

Fig. S10 Correlation analysis for climatic sensitivity traits ring density and monthly mean temperature.

Table S1 Summary of provenances location, mean annual temperature of provenance, mean annual precipitation and number of sampled trees per provenance.

Table S2 Spearman rank correlations between monthly climate variables.

Table S3 Detailed results of the Genotype Phenotype Association (GPA) analysis with TASSEL.

Table S4 Detailed results of the Genotype Phenotype Association (GPA) analysis with TASSEL when excluding the two populations from the southern group.

Table S5 Comparisons between the number of SNPs detected in GPAs with TASSEL between candidate and non-candidate SNPs.

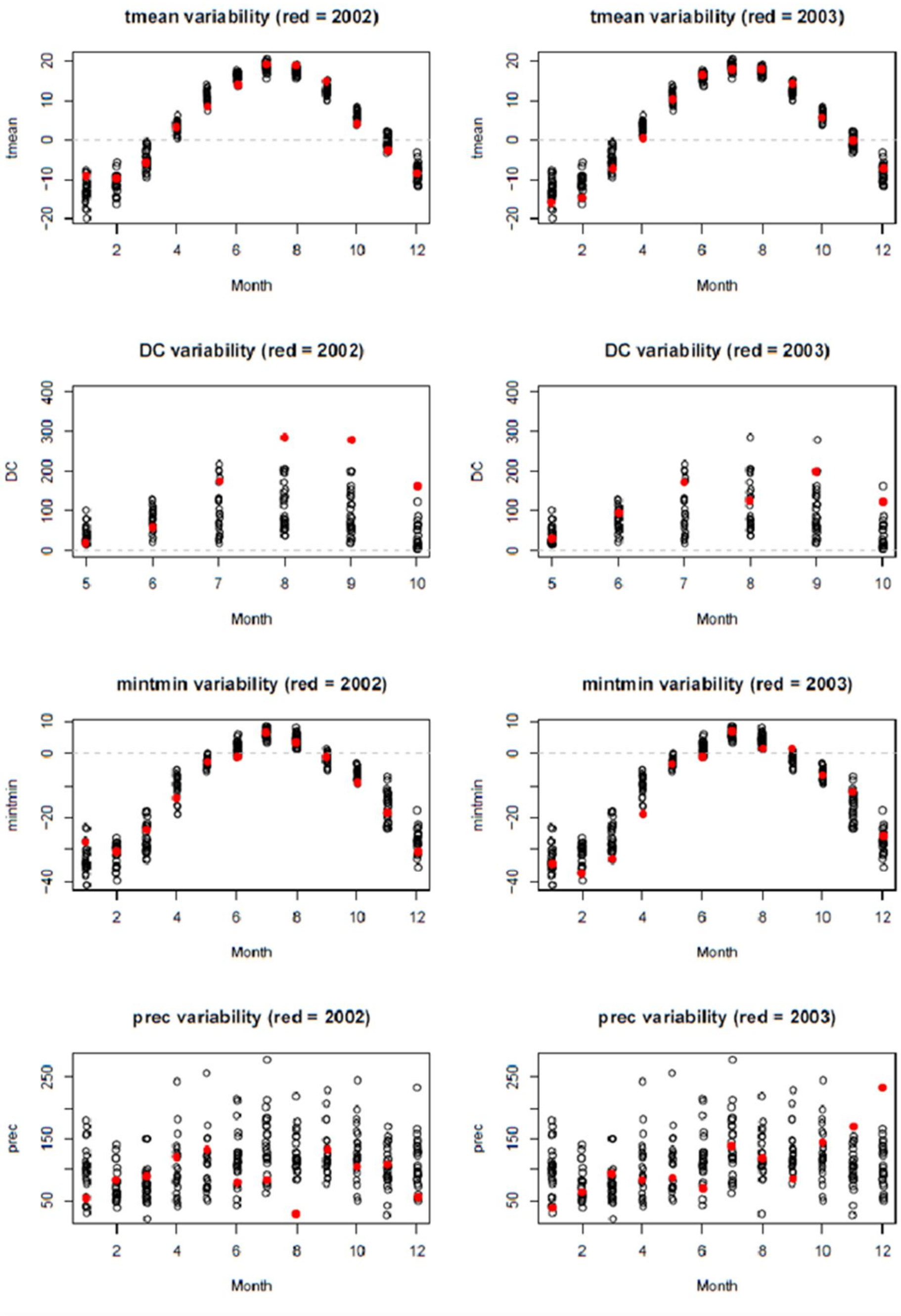

Fig. S1 Monthly mean temperature (tmean), drought code (DC), minimum temperature (mintmin) and precipitation (prec) in 2002 and 2003 (red point) at Valcartier, compared to inter-annual variability during the 1993-2014 period (black points).

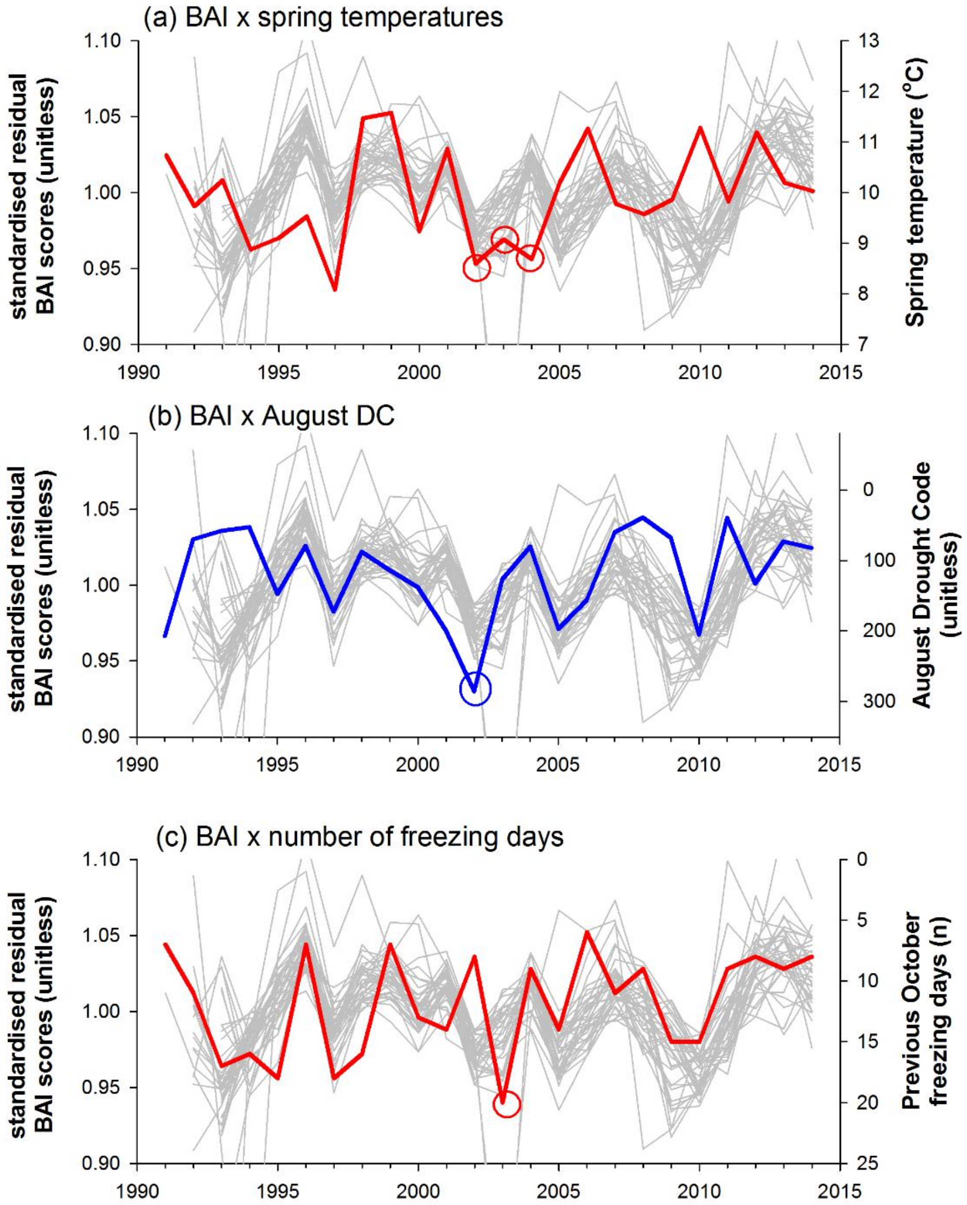

Fig. S2 (a) Spring (April-May) mean temperature and (b) August drought code (DC), and (c) number of days with minimum temperature below zero at Valcartier, compared to inter-annual variability in standardized residual BAI scores (grey lines). Note the inverted y-scale in b) and c). Drought Code is unitless and ranges from 0 to about 800, with low values indicating low drought risk and high values indicating high drought risk. Anomalies of 2002-2004 are circled.

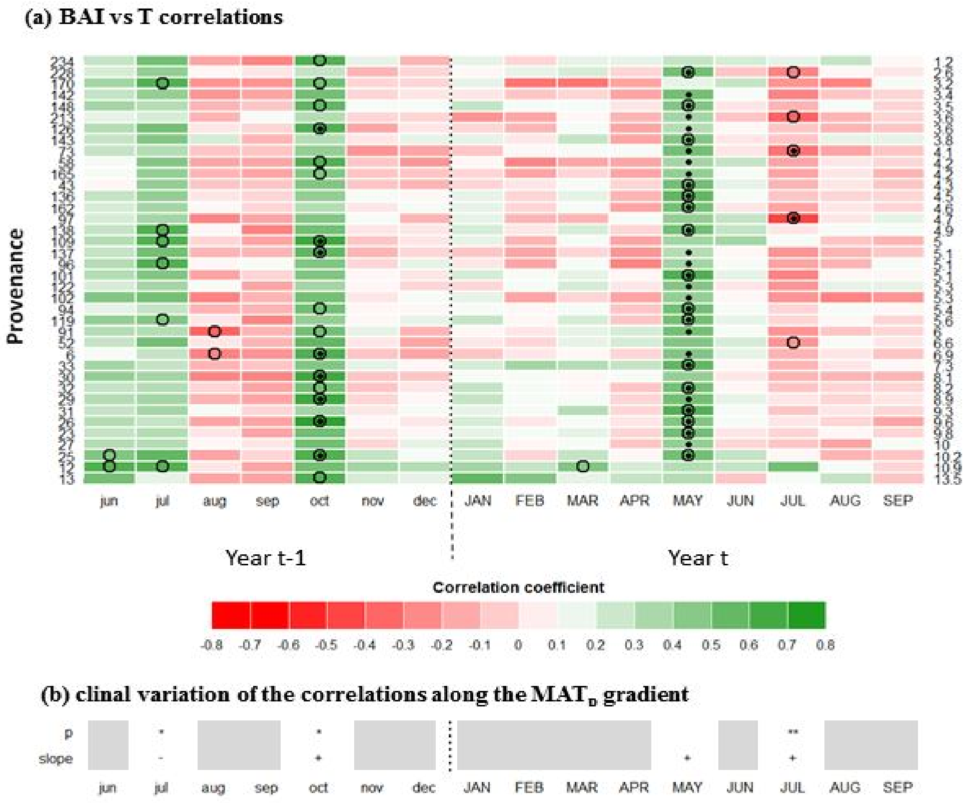

Fig. S3 Correlation analysis for climatic sensitivity (*CS*) traits basal area increment (BAI) and monthly temperature (T). This figure is the same as in Fig. 4 but with the addition of the significant coefficients for the bootstrapped response function method, indicated by dots. Analyses were computed between *P. strobus* standardized residuals of BAI scores (Fig. 3b) and monthly mean temperature at the Valcartier common garden experiment (Quebec, Canada) over the 1993–2014 period. Populations (rows) are ordered by increasing mean annual temperature of the provenance (MAT_p; right axis labels). Months in capital letters represent the current year of ring formation; months in lower case represent climate variables during the year preceding ring formation. Significant coefficients are represented by circles. (**b**) Test for clinal variations in correlation coefficients along the MAT_p gradient: *p* value and slope sign of a regression against MAT_p. Significance codes: •: $p < 0.1$; *: $p < 0.05$; **: $p < 0.01$; ***: $p < 0.001$. The response function analysis give credence to the correlation analysis by indicating significant positive relationship between standardised residual BAI scores and October temperature the year previous to ring formation and May temperature of the year current to ring formation.

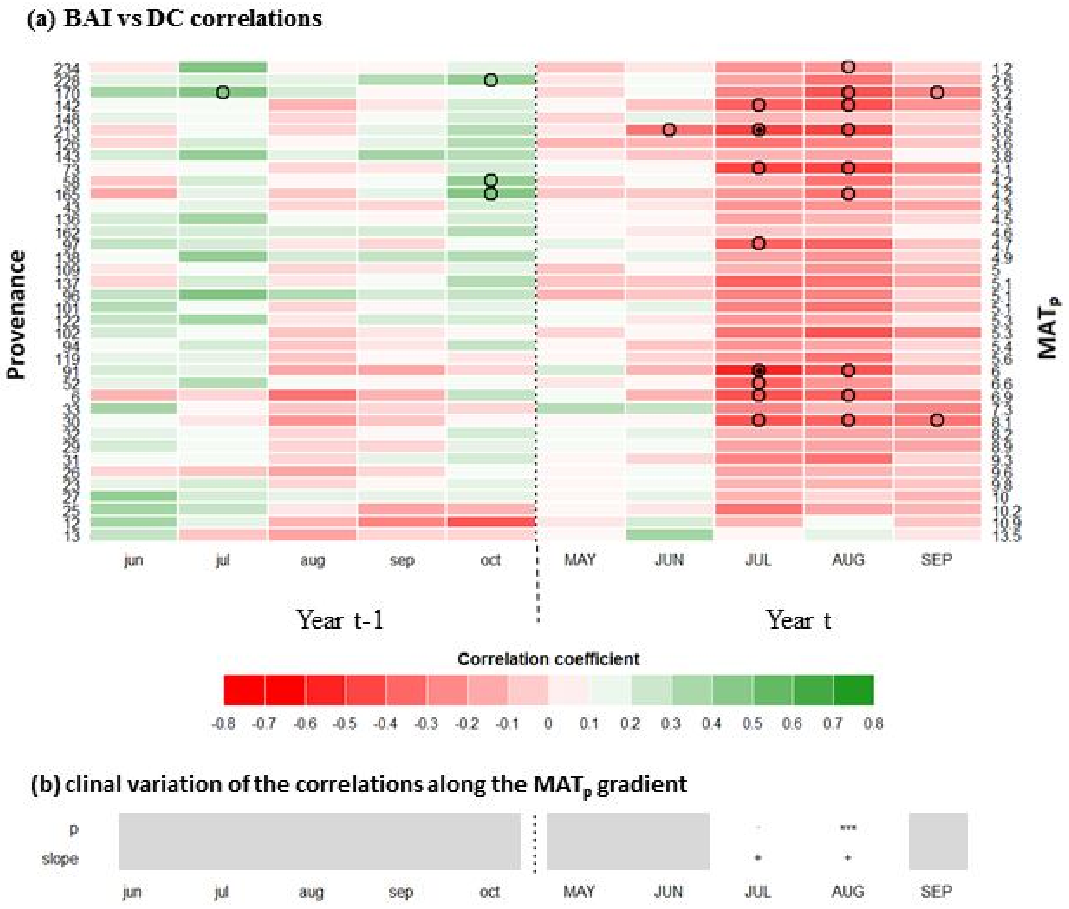

Fig. S4 Correlation analysis for climatic sensitivity (*CS*) traits basal area increment (BAI) and monthly drought code (DC). This figure is the same as in Fig. 5 but with the addition of the significant coefficients for the bootstrapped response function method, indicated by dots. Analyses were computed between *P. strobus* standardized residuals of BAI scores (Fig. 3b) and monthly DC at the Valcartier common garden experiment (Quebec, Canada) over the 1993–2014 period. Populations (rows) are ordered by increasing mean annual temperature of the provenance (MAT_p; right axis labels). Months in capital letters represent the current year of ring formation; months in lower case represent climate variables during the year preceding ring formation. Significant coefficients are represented by circles. (**b**) Test for clinal variations in correlation coefficients along the MAT_p gradient: *p* value and slope sign of a regression against MAT_p. Significance codes: •: $p < 0.1$; *: $p < 0.05$; **: $p < 0.01$; ***: $p < 0.001$.

Fig. S5 (*Next two pages*). Genetic structure assessed through a discriminant analysis of principal components (DAPC, Jombart *et al.* 2010): **(a)** BIC versus the number of clusters in successive k-means clustering analysis, **(b)** barplot of membership probabilities for k = 3 clusters (each bar represent an individual tree), **(c)** geographic map of populations' membership probabilities for k = 3 clusters. To estimate population structure (Q-matrix), we ran DAPC using the 128 genotyped SNPs. In a preliminary DAPC analysis, we found that a weaker structure was detected when we excluded the 21 candidate SNPs from Nadeau et al. (2016) since these SNPs have higher F_{ST} (F_{ST} = 0.074, H_O = 0.311) as compared to non-candidate SNPs (F_{ST} = 0.040, H_O = 0.329). This was expected because these SNPs showed the highest among-population differentiation in F_{ST} outlier and GEA tests (Nadeau et al. 2016). Because we cannot exclude the hypothesis that a proportion of the candidate SNPs are false positives, we chose the more conservative approach of including them in DAPC analyses to avoid under correcting for population structure in TASSEL models. To obtain better estimates of cluster membership probabilities in DAPC analyses, we combined the 225 genotyped trees from this study with 821 additional trees (133 provenances) sampled across the entire range and genotyped on the same set of SNPs (Nadeau *et al.* 2015). SNP data for the 1046 individuals was first transformed into principal components (PCs) to satisfy DAPC requirements of uncorrelated variables. The first 40 PCs that explained more than 1% of the variation (total variation explained: 59%) were included in DAPC analyses (varying the number of PCs did not affect the results). Successive k-means clustering from k = 1 to k = 10 clusters suggested that k = 5 to k= 7 clusters best explained the data (Figure S2A, lowest value of the Bayesian Information Criterion, BIC). However, for k = 4 or higher, the clusters were highly admixed and could not be clearly geographically delimited (not shown). Thus, we used the membership probabilities for k = 3 clusters (Figs. S3B and S3C) as the Q matrix to correct for population structure in TASSEL analyses. The 3 clusters detected are similar to previous results from Nadeau et al. (2015) using the software STRUCTURE. The red cluster represents the southern group, and the blue and green clusters represent the northern group with a weak pattern of differentiation from west to east (individuals within the northern group are ordered by longitude in Fig. S3B).

(a)

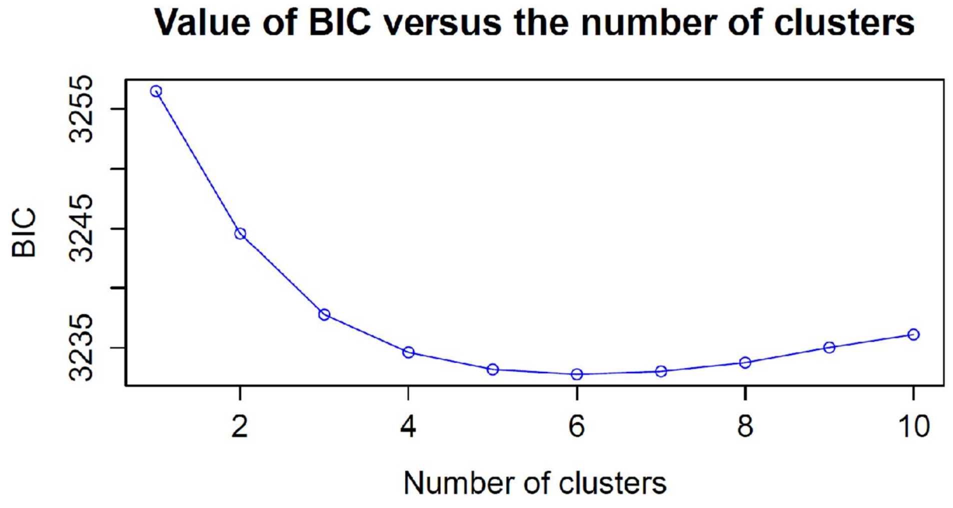
Value of BIC versus the number of clusters
BIC
3235
3245
3255
2
4
6
8
10
Number of clusters

(b)

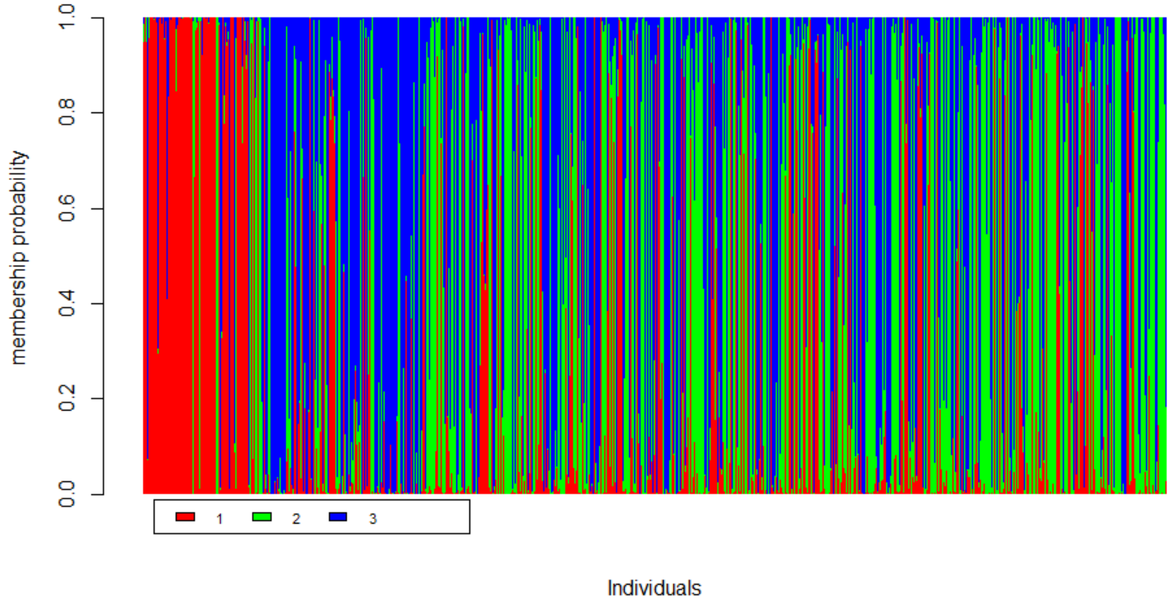
membership probability
0.0
0.2
0.4
0.6
0.8
1.0
1
2
3
Individuals

(c)

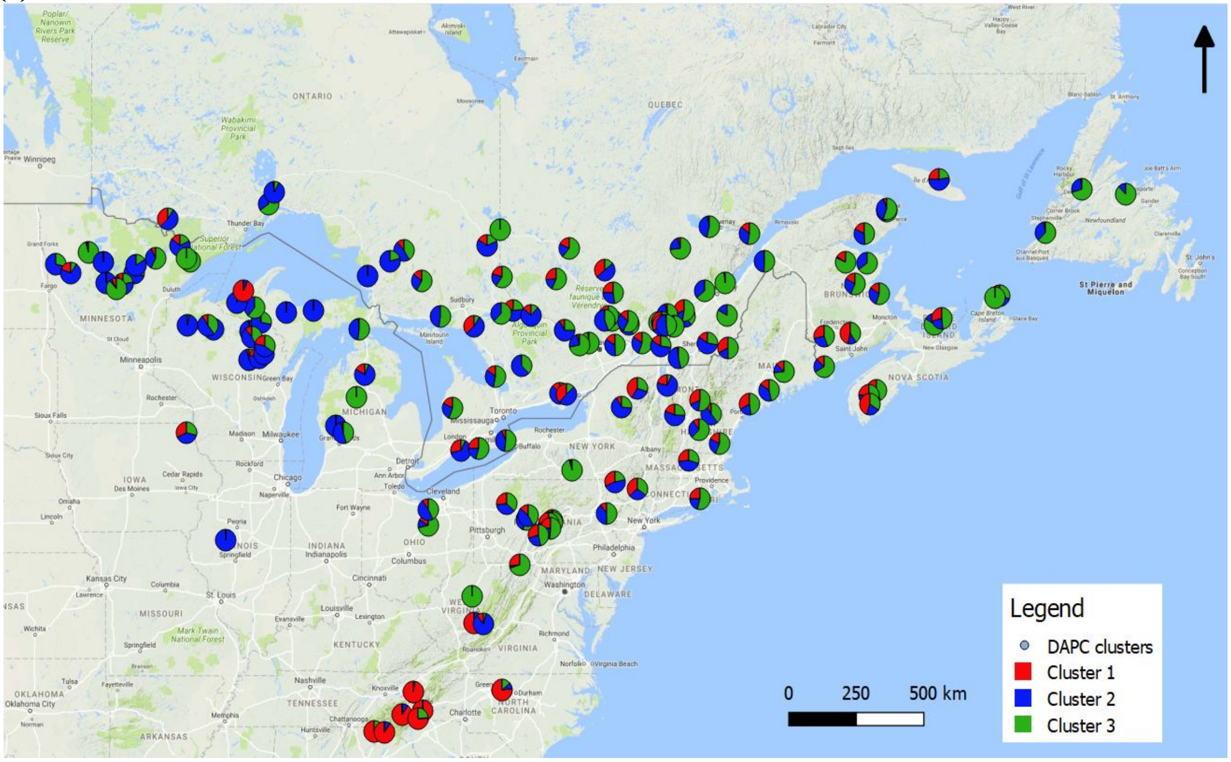

Legend
DAPC clusters
Cluster 1
Cluster 2
Cluster 3
0
250
500 km

Fig. S6 (*Next page*) Correlation analysis for climatic sensitivity (*CS*) traits basal area increment (BAI) and monthly number of days below 0 ºC (freezing days). (**a**) Bootstrapped correlation coefficients were computed between *Pinus strobus* BAI residual chronologies and the monthly number of freezing days at Valcartier common garden plantation (Québec, Canada) for the period 1993-2014. Provenances (rows) are ordered by increasing mean annual temperature of provenance (MAT_p; right axis labels). Months in capital letters represent the current year of ring formation; months in lower case represent climate variables during the year preceding ring formation. Significant correlations are represented by dots for the bootstrapped principal component regression method and by circles for the bootstrapped correlation method. (**b**) Test for clinal variations in the correlation coefficients along the MAT_p gradient: *p* value and slope sign of a regression against MAT_p. Significativity codes: "." $p<0.1$; " * ": $p<0.05$; " ** ": $p<0.01$; " *** ": $p<0.001$.

(a) BAI-freezing days correlations

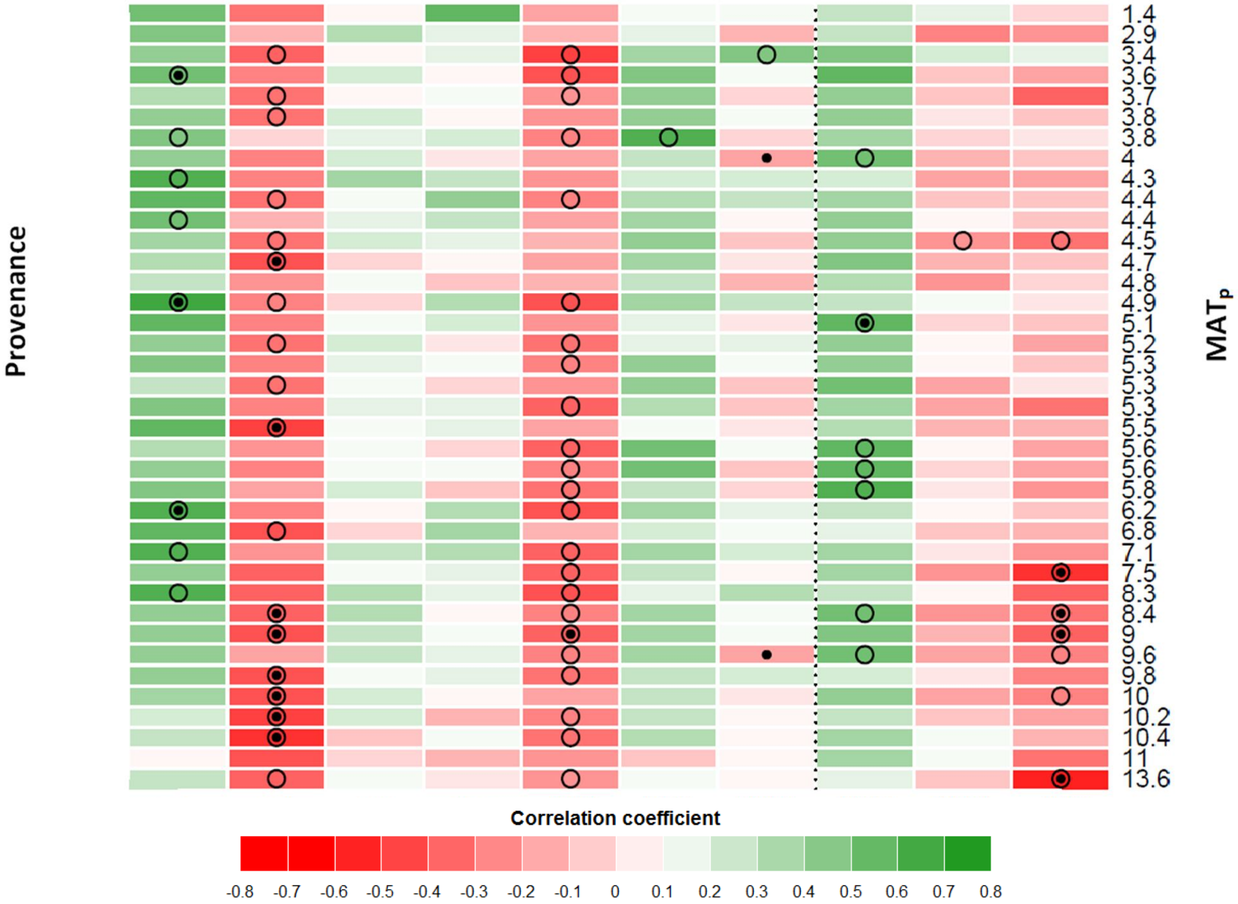

(b) clinal variation of the correlations along the MAT_p gradient

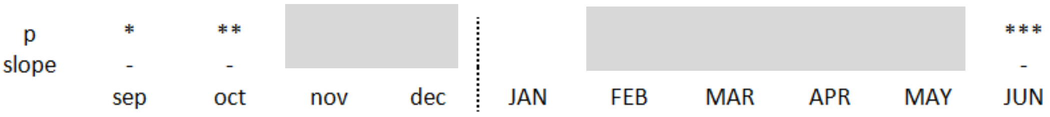

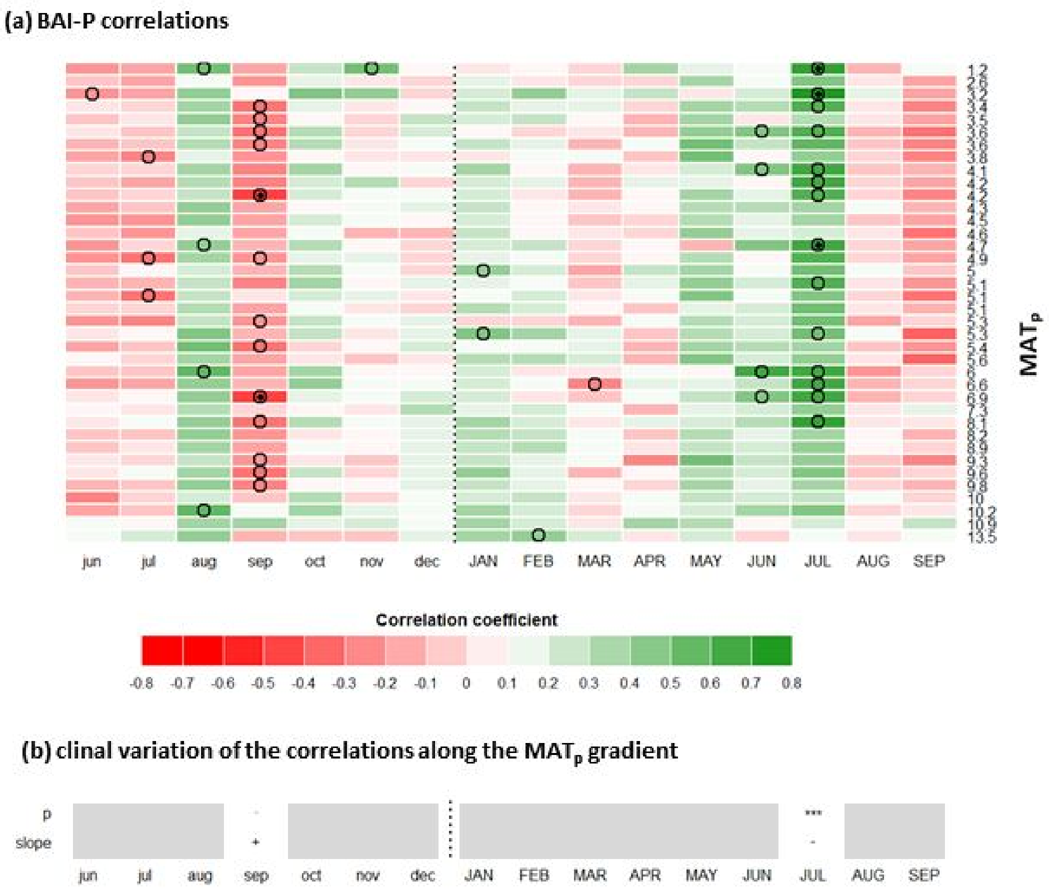

Fig. S7 Correlation analysis for climatic sensitivity (*CS*) traits basal area increment (BAI) and monthly precipitation. (**a**) Bootstrapped correlation coefficients were computed between *Pinus strobus* BAI residual chronologies and the monthly mean precipitation at Valcartier common garden plantation (Québec, Canada) for the period 1994-2014. Provenances (rows) are ordered by increasing mean annual temperature of provenance (MAT_p; right axis labels). Months in capital letters represent the current year of ring formation; months in lower case represent climate variables during the year preceding ring formation. Significant correlations are represented by dots for the bootstrapped principal component regression method and by circles for the bootstrapped correlation method. (**b**) Test for clinal variations in the correlation coefficients along the MAT_p gradient: *p* value and slope sign of a regression against MAT_p. Significativity codes: "." p<0.1; " * ": p<0.05; " ** ": p<0.01; " *** ": p<0.001.

Fig. S8 (*Next two pages*) Comparison of growth-climate relationships at the provenance and at the individual levels. Bootstrapped correlation coefficients were computed between *Pinus strobus* basal area increment residual chronologies and the monthly climatic variables at Valcartier common garden plantation (Québec, Canada) for the period 1993-2014. Provenances or individuals (rows) are ordered by increasing mean annual temperature of provenance (MAT_p; right axis labels). Months in capital letters represent the current year of ring formation; months in lower case represent climate variables during the year preceding ring formation. Significant correlations are represented by dots for the bootstrapped principal component regression method and by circles for the bootstrapped correlation method.

Population level (one row per provenance) **Individual level (one row per individual)**

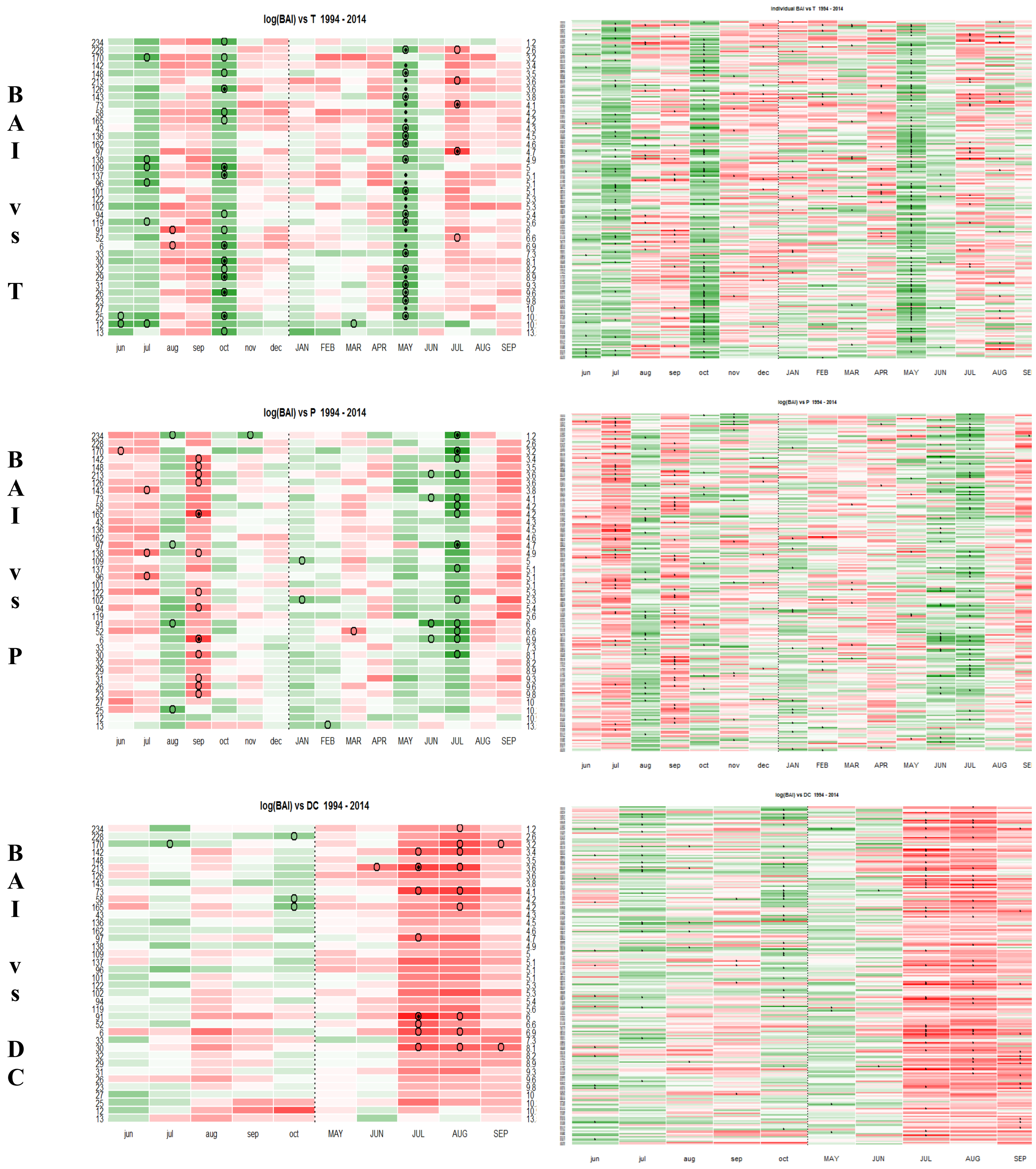

BAI vs Tmin

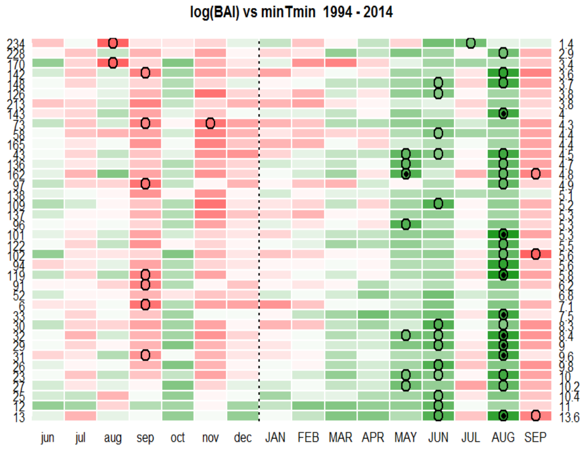

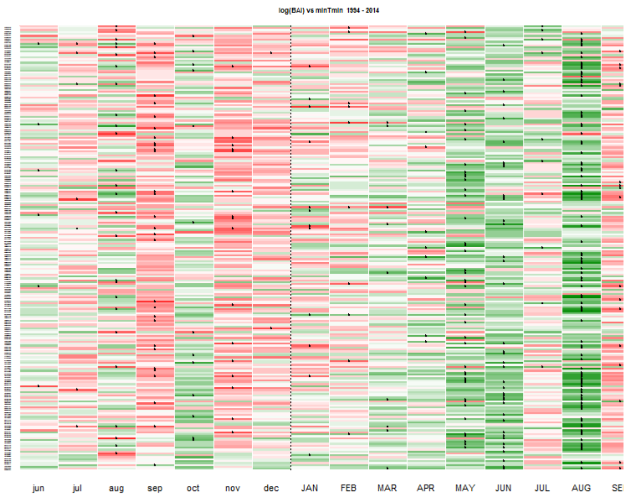

BAI vs nFrostdays

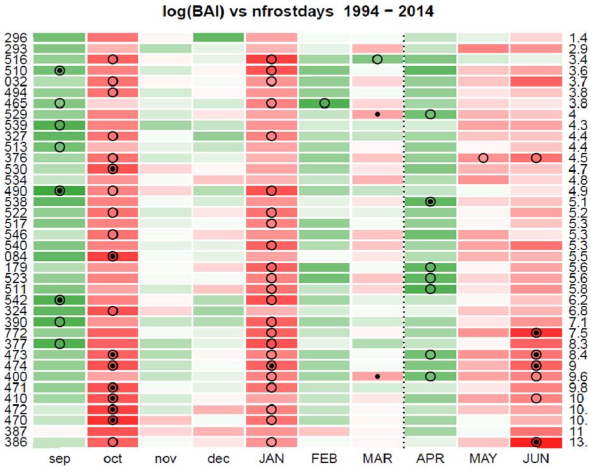

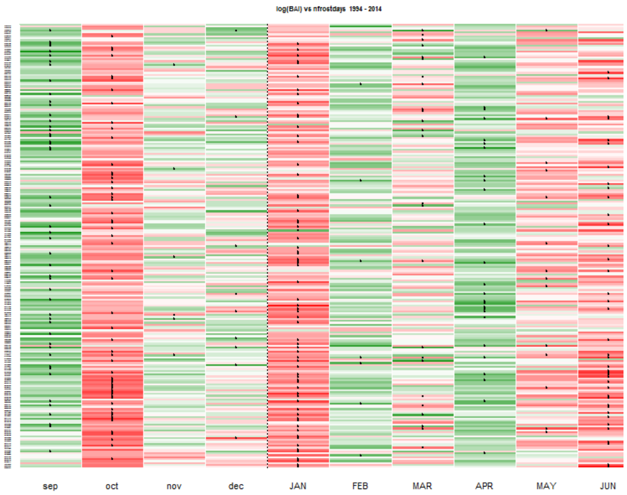

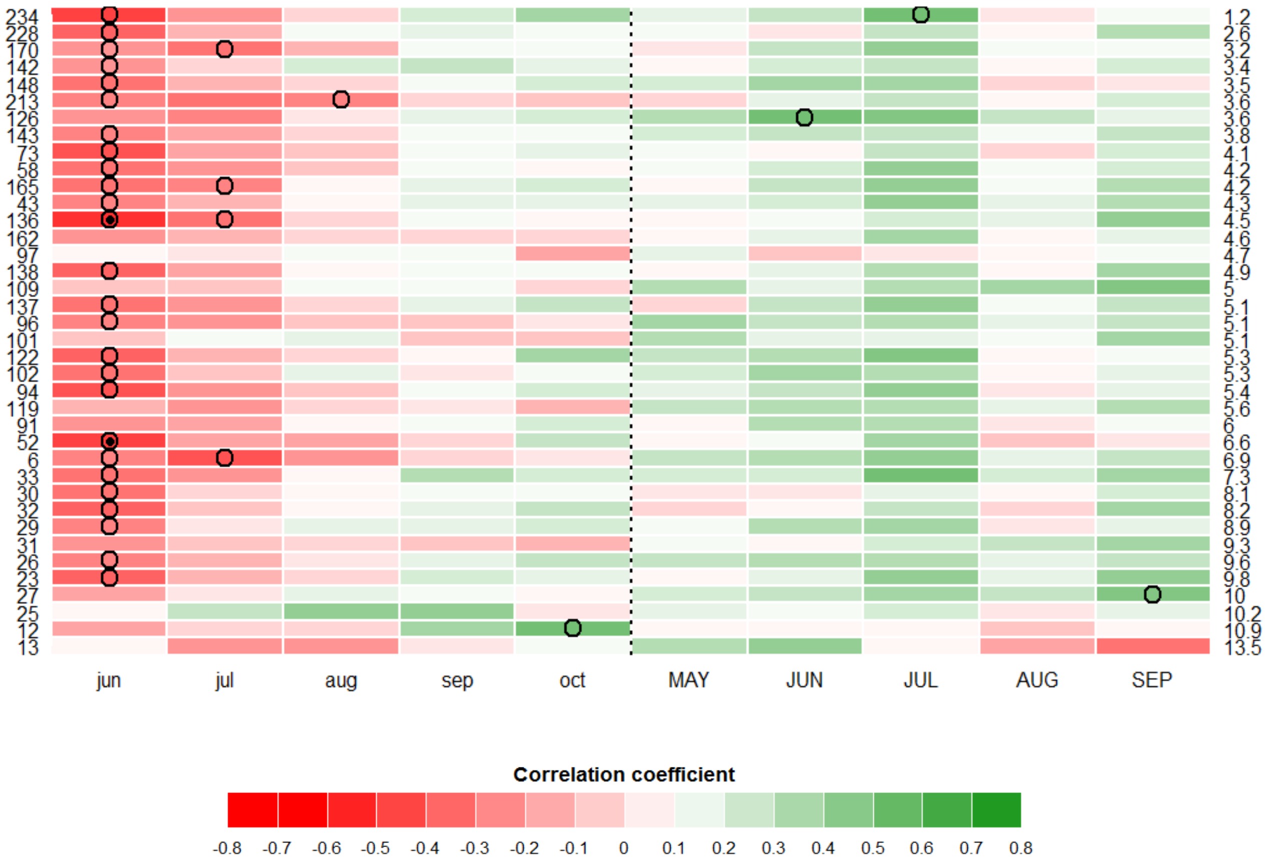

Fig. S9 Correlation analysis for climatic sensitivity (*CS*) traits ring average density and monthly drought code (DC). Bootstrapped correlation coefficients were computed between *Pinus strobus* mean annual density residual chronologies and the monthly drought code at Valcartier common garden plantation (Québec, Canada) for the period 1994-2014. Provenances (rows) are ordered by increasing mean annual temperature of provenance (MAT_p; right axis labels). Months in capital letters represent the current year of ring formation; months in lower case represent climate variables during the year preceding ring formation. Significant correlations are represented by dots for the bootstrapped principal component regression method and by circles for the bootstrapped correlation method.

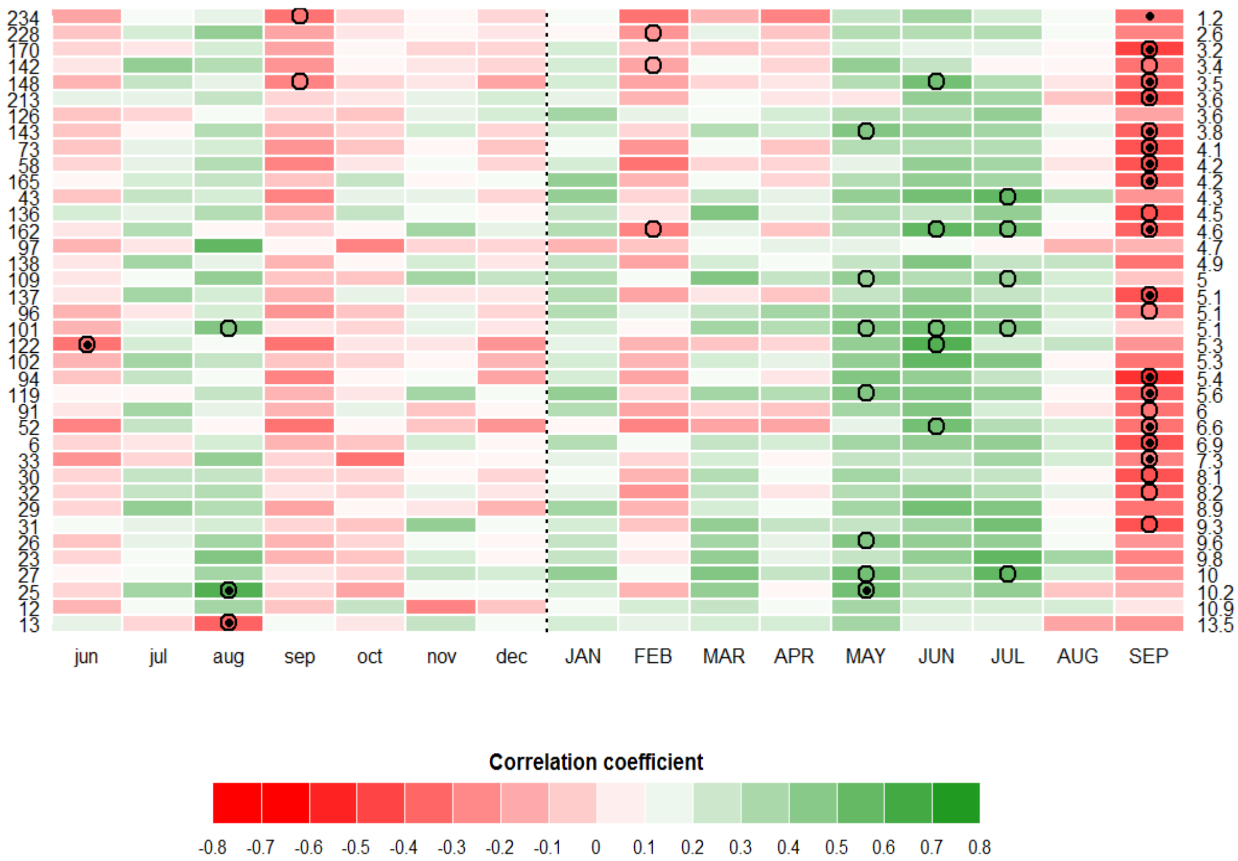

Fig. S10 Correlation analysis for climatic sensitivity (*CS*) traits ring density and monthly mean temperature. Bootstrapped correlation coefficients were computed between *Pinus strobus* mean annual density residual chronologies and the monthly mean temperature at Valcartier common garden plantation (Québec, Canada) for the period 1993-2014. Provenances (rows) are ordered by increasing mean annual temperature of provenance (MAT_p; right axis labels). Months in capital letters represent the current year of ring formation; months in lower case represent climate variables during the year preceding ring formation. Significant correlations are represented by dots for the bootstrapped principal component regression method and by circles for the bootstrapped correlation method.

Table S1 Summary of provenances location, mean annual temperature of provenance (MATp), mean annual precipitation (MAPp) and number of sampled trees per provenance.

Id	Province/State	Provenance name	Latitude	Longitude	Altitude (m a.s.l.)	MATp (°C)	MAPp (mm)	Number
234	ONTARIO	MACDIARMID	49.16	-88.09	261	1.41	839	5
228	ONTARIO	ATIKOKAN	48.38	-92.14	392	2.86	756	5
170	QUEBEC	LAC BEAUCHENE	46.40	-79.00	349	3.41	1009	5
142	QUEBEC	CANTON RIVARD	46.15	-75.20	251	3.63	1011	6
148	QUEBEC	DEUX RIVIERES	46.16	-78.18	311	3.72	879	10
126	QUEBEC	LAC LABELLE	46.05	-74.55	255	3.84	1075	5
213	MINNESOTA	MARCELL	47.35	-94.40	425	3.84	689	6
143	QUEBEC	LAC 31 MILLES	46.15	-75.50	229	3.98	1003	6
73	QUEBEC	BISHOPTON	45.35	-71.33	394	4.27	1190	7
58	ONTARIO	MINDEN 1	45.00	-78.40	396	4.42	1039	6
165	QUEBEC	CANTON DE CALONNE	46.30	-73.10	94	4.43	1022	7
43	NEW YORK	ADIRONDACK MOUNTAINS	43.56	-74.50	603	4.53	1279	8
136	QUEBEC	PEPINIERE GRANDES PILES	46.40	-72.45	26	4.68	1041	7
162	QUEBEC	ST ETIENNE DES GRES	46.25	-72.40	34	4.78	1012	6
97	QUEBEC	ILE AUX ALLUMETTES	45.54	-77.01	151	4.89	807	5
138	QUEBEC	ST ALPHONSE DE RODRIGUEZ	46.10	-73.40	76	5.13	1006	6
109	QUEBEC	ST GERARD D YAMASKA	46.00	-72.50	62	5.20	1038	5
137	QUEBEC	ST-NORBERT	46.10	-73.30	40	5.30	997	7
96	QUEBEC	DAVIDSON	45.53	-76.46	116	5.34	842	6
101	QUEBEC	MONT-ROLLAND	45.56	-74.07	75	5.34	1039	6
122	QUEBEC	JOLIETTE	46.03	-73.27	23	5.45	997	7
102	QUEBEC	LAC DANFORD	45.56	-76.09	109	5.56	893	4
94	QUEBEC	LAC VERT	45.51	-75.08	62	5.64	965	7
119	QUEBEC	STE PHILOMENE	46.02	-73.11	18	5.82	978	7
91	QUEBEC	ST EPIPHANIE	45.50	-73.33	22	6.17	1019	7
52	ONTARIO	TWEED	44.29	-77.19	140	6.75	915	6
6	MAINE	SEARSMONT	44.36	-69.20	65	7.09	1217	7
33	CONNECTICUT	YALE UNION FOREST EASTFORD	41.55	-75.05	314	7.51	1066	4
30	PENNSYLVANIA	CLEARFIELD	41.01	-78.26	459	8.28	1019	7
32	PENNSYLVANIA	COOKSBURG	41.20	-79.15	474	8.38	1137	8
29	PENNSYLVANIA	MADERA	40.55	-78.30	428	9.04	1033	6
31	OHIO	OHIO FINDLEY STATE PARK	41.10	-82.15	291	9.56	950	6
26	PENNSYLVANIA	POTTERS MILLS	40.48	-77.38	529	9.76	1204	6
23	PENNSYLVANI	HUNTINGTON & WARREN CO	40.29	-78.01	378	10.02	1012	7

	A							
27	PENNSYLVANIA	SWAMP HOUSE HOLLOW	40.50	-77.30	274	10.17	1069	6
25	PENNSYLVANIA	VIRA	40.40	-77.33	253	10.38	1060	8
12	NORTH CAROLINA	JACKSON CO 687	35.20	-83.15	1089	11.03	1994	2
13	NORTH CAROLINA	BUNCOMBE	35.30	-82.30	524	13.60	1493	2

Table S2 Spearman rank correlations (r) between monthly climate variables. The period of analysis is 1993-2014 (n = 22 years). Abbreviations of climatic variables: DC = Drought Code, T = monthly means of daily mean temperatures, P = monthly precipitation totals. Abbreviations for months: 1 = January, 2 = February, 3 = March, …, 12 = December. ns = $-0.423 < r < +0.423$. Shaded areas highlight the analysis between DC and P and T during summer months.

Variable	DC4	DC5	DC6	DC7	DC8	DC9	DC10	T1	T2	T3	T4	T5	T6	T7	T8	T9	T10	T11	T12	P1	P2	P3	P4	P5	P6	P7	P8	P9	P10	P11	P12
**DC4**	1.00																														
**DC5**	0.62	1.00																													
**DC6**	ns	0.46	1.00																												
**DC7**	ns	ns	0.49	1.00																											
**DC8**	ns	ns	ns	0.71	1.00																										
**DC9**	ns	ns	ns	ns	0.58	1.00																									
**DC10**	ns	-0.55	-0.46	ns	ns	ns	1.00																								
**T1**	ns	ns	ns	0.43	ns	ns	ns	1.00																							
**T2**	ns	ns	ns	ns	ns	ns	ns	ns	1.00																						
**T3**	ns	ns	ns	ns	ns	ns	ns	ns	0.62	1.00																					
**T4**	ns	ns	ns	ns	ns	ns	-0.50	ns	0.62	0.43	1.00																				
**T5**	0.63	0.47	ns	ns	ns	ns	-0.44	ns	0.44	ns	ns	1.00																			
**T6**	ns	0.60	ns	ns	ns	ns	ns	ns	ns	ns	ns	ns	1.00																		
**T7**	ns	ns	ns	ns	ns	ns	ns	0.47	ns	0.47	ns	ns	ns	1.00																	
**T8**	ns	ns	ns	0.50	ns	ns	ns	ns	ns	ns	ns	ns	ns	ns	1.00																
**T9**	ns	ns	ns	ns	ns	ns	ns	ns	0.46	ns	ns	ns	ns	ns	ns	1.00															
**T10**	ns	ns	ns	ns	ns	ns	ns	ns	ns	ns	ns	ns	ns	ns	ns	ns	1.00														
**T11**	ns	ns	ns	ns	ns	ns	ns	ns	ns	ns	ns	ns	ns	ns	ns	ns	ns	1.00													
**T12**	ns	0.56	0.52	ns	ns	ns	-0.43	ns	ns	ns	ns	ns	0.65	ns	ns	ns	ns	ns	1.00												
**P1**	ns	ns	ns	ns	ns	ns	ns	ns	ns	ns	ns	ns	ns	ns	-0.47	-0.69	ns	ns	ns	1.00											
**P2**	-0.66	ns	ns	ns	ns	ns	ns	ns	ns	ns	ns	ns	ns	ns	ns	ns	ns	ns	ns	ns	1.00										
**P3**	ns	ns	ns	ns	ns	ns	ns	ns	ns	ns	ns	ns	ns	ns	ns	ns	ns	ns	ns	ns	ns	1.00									
**P4**	ns	ns	ns	ns	ns	ns	ns	ns	ns	ns	ns	-0.44	ns	ns	ns	ns	ns	ns	ns	ns	ns	ns	1.00								
**P5**	ns	-0.82	-0.55	ns	ns	ns	0.54	ns	ns	ns	ns	ns	ns	ns	ns	ns	ns	ns	-0.43	ns	ns	ns	ns	1.00							
**P6**	ns	ns	-0.48	-0.74	ns	ns	ns	ns	ns	ns	ns	ns	ns	ns	ns	ns	ns	ns	ns	ns	ns	ns	ns	ns	1.00						
**P7**	ns	ns	ns	-0.70	-0.79	-0.57	ns	ns	ns	ns	ns	ns	ns	ns	-0.52	ns	ns	ns	ns	ns	ns	ns	ns	ns	ns	1.00					
**P8**	ns	ns	ns	ns	ns	-0.60	ns	ns	ns	ns	ns	ns	ns	ns	ns	ns	ns	ns	ns	ns	ns	ns	ns	ns	ns	ns	1.00				
**P9**	ns	ns	ns	ns	ns	ns	-0.72	ns	ns	ns	0.53	ns	ns	ns	ns	ns	ns	ns	ns	ns	ns	ns	ns	-0.44	ns	ns	ns	1.00			
**P10**	ns	ns	ns	ns	ns	ns	ns	ns	ns	ns	ns	0.51	0.56	ns	ns	ns	ns	ns	ns	ns	ns	ns	ns	ns	ns	ns	ns	ns	1.00		
**P11**	ns	ns	ns	ns	ns	ns	ns	ns	ns	ns	ns	ns	ns	ns	ns	ns	ns	ns	ns	ns	ns	ns	ns	ns	ns	ns	ns	ns	ns	1.00	
**P12**	ns	ns	ns	ns	-0.45	ns	ns	ns	ns	ns	ns	ns	ns	ns	ns	ns	ns	ns	ns	ns	ns	ns	ns	ns	ns	ns	ns	ns	ns	ns	1.00

Table S3 Detailed results of the Genotype Phenotype Association (GPA) analysis with TASSEL (n = 225 trees). For each SNP-trait combinations, p values and marker R2 are reported. For each trait, three models were tested (1) no correction for population structure; (2) correction for population structure (Q model); and (3) correction for population structure and kinship (Q + K model).

See accompanying Excel file

Table S4 Detailed results of the Genotype Phenotype Association (GPA) analysis with TASSEL when excluding the two populations from the southern group (n = 221 trees).

See accompanying Excel file

Table S5 Comparisons between the number of SNPs detected in GPAs with TASSEL (n = 225 trees) between candidate SNPs (21) and non-candidate SNPs (107).

		Candidate SNPs (21)		Non-candidates SNPs (107)	
Trait	Significance	Number of SNPs	Percentage of SNPs tested (out of 21)	Number of SNPs	Percentage of SNPs tested (out of 107)
DBH_{21}	uncertain	1	4.762	2	1.869
	likely	1	4.762	0	0.000
	very likely	0	0.000	6	5.607
	Total	2	9.524	8	7.477
H_{21}	uncertain	0	0.000	0	0.000
	likely	1	4.762	0	0.000
	very likely	0	0.000	8	7.477
	Total	1	4.762	8	7.477
$MeanBAI_{1993-2014}$	uncertain	0	0.000	0	0.000
	likely	0	0.000	1	0.935
	very likely	1	4.762	5	4.673
	Total	1	4.762	6	5.607
BAI2003	uncertain	2	9.524	1	0.935
	likely	0	0.000	0	0.000
	very likely	3	14.286	5	4.673
	Total	5	23.810	6	5.607
CS_{BAI}-Temp.$Jul_{(t)}$	uncertain	0	0.000	6	5.607
	likely	1	4.762	2	1.869
	very likely	2	9.524	3	2.804
	Total	3	14.286	11	10.280
CS_{BAI}-Prec.$Jul_{(t)}$	uncertain	1	4.762	0	0.000
	likely	0	0.000	0	0.000
	very likely	1	4.762	7	6.542
	Total	2	9.524	7	6.542
CS_{BAI}-Drought.$Aug_{(t)}$	uncertain	1	4.762	1	0.935
	likely	1	4.762	1	0.935
	very likely	1	4.762	4	3.738
	Total	3	14.286	6	5.607
CS_{BAI}-Freeze.$Oct_{(t-1)}$	uncertain	2	9.524	1	0.935
	likely	1	4.762	2	1.869
	very likely	0	0.000	7	6.542
	Total	3	14.286	10	9.346

Supplementary Table 5 (*continued*)

		Candidate SNPs (21)		Non-candidates SNPs (107)	
Trait	Significance	Number of SNPs	Percentage of SNPs tested (out of 21)	Number of SNPs	Percentage of SNPs tested (out of 107)
CS_{BAI}-Freeze.$Jun_{(t)}$	uncertain	2	9.524	4	3.738
	likely	2	9.524	6	5.607
	very likely	0	0.000	0	0.000
	Total	4	19.048	10	9.346

References

Jombart T, Devillard S, Balloux F. **2010**. Discriminant analysis of principal components: a new method for the analysis of genetically structured populations. *BMC Genetics* **11**: 94.

Nadeau S, Godbout J, Lamothe M, Gros-Louis M-C, Isabel N, Ritland K. **2015**. Contrasting patterns of genetic diversity across the ranges of *Pinus monticola* and *P. strobus*: A comparison between eastern and western North American postglacial colonization histories. *American Journal of Botany* **102**: 1342–1355.

Nadeau S, Meirmans PG, Aitken SN, Ritland K, Isabel N. **2016**. The challenge of separating signatures of local adaptation from those of isolation by distance and colonization history: The case of two white pines. *Ecology and Evolution* **6**: 8649–8664.
